# Prediction by Promoter Logic in Bacterial Quorum Sensing

**DOI:** 10.1371/journal.pcbi.1002361

**Published:** 2012-01-19

**Authors:** Navneet Rai, Rajat Anand, Krishna Ramkumar, Varun Sreenivasan, Sugat Dabholkar, K. V. Venkatesh, Mukund Thattai

**Affiliations:** 1 National Centre for Biological Sciences, Tata Institute of Fundamental Research, UAS/GKVK Campus, Bangalore, India; 2 Department of BioSciences and Bioengineering, Indian Institute of Technology Bombay, Powai, Mumbai, India; 3 Department of Chemistry, Indian Institute of Technology Bombay, Powai, Mumbai, India; 4 Department of Biological Sciences, St. Xavier's College, Mumbai, India; Duke Institute for Genome Sciences & Policy, United States of America

## Abstract

Quorum-sensing systems mediate chemical communication between bacterial cells, coordinating cell-density-dependent processes like biofilm formation and virulence-factor expression. In the proteobacterial LuxI/LuxR quorum sensing paradigm, a signaling molecule generated by an enzyme (LuxI) diffuses between cells and allosterically stimulates a transcriptional regulator (LuxR) to activate its cognate promoter (pR). By expressing either LuxI or LuxR in positive feedback from pR, these versatile systems can generate smooth (monostable) or abrupt (bistable) density-dependent responses to suit the ecological context. Here we combine theory and experiment to demonstrate that the promoter logic of pR – its measured activity as a function of LuxI and LuxR levels – contains all the biochemical information required to quantitatively predict the responses of such feedback loops. The interplay of promoter logic with feedback topology underlies the versatility of the LuxI/LuxR paradigm: LuxR and LuxI positive-feedback systems show dramatically different responses, while a dual positive/negative-feedback system displays synchronized oscillations. These results highlight the dual utility of promoter logic: to probe microscopic parameters and predict macroscopic phenotype.

## Author Summary

Bacterial cells constantly communicate with one another by exchanging chemical signals, which constitute a rich source of information about the proximity of friends or foes in the environment. These signals can be used to coordinate the actions of cells across a population. For example, pathogenic bacteria infecting a host can remain quiescent, only becoming virulent once they attain a sufficient cell density. Such coordination, regulated by so-called quorum-sensing systems, works on the following principle: every cell in the population secretes a specific chemical signal; the more cells there are, the more signal is generated; when individual cells sense that the signal has crossed some threshold, they launch a response. The nature of the response depends on the detailed molecular wiring of the secretion and sensing system, which can vary from species to species. It is often impossible to determine all these molecular details for any given system. Borrowing ideas from control theory, we show that this internal wiring can be largely ignored, and these systems can be considered as ‘black boxes’. Our experiments demonstrate that the measured input-output logic of the black box, which we term ‘promoter logic’, is sufficient to predict the diverse responses of different quorum-sensing systems.

## Introduction

Free-living bacteria use quorum-sensing systems – dedicated chemical communication channels – to coordinate population-wide behaviors [1], [2]. These systems regulate the cell-density-dependence of several bacterial activities, including bioluminescence, competence and sporulation, biofilm formation, and virulence factor expression [3], [4]. In many gram-negative bacteria, quorum sensing is mediated by two key proteins termed LuxI and LuxR, and a class of signaling molecules known as acyl-homoserine lactones (AHLs) [1]. LuxI is the enzyme that synthesizes AHL, with the LuxI homologs of different species generating distinct AHL side-chain variants; LuxR, when bound to its cognate AHL, functions as a transcriptional activator. The AHL generated within each cell freely diffuses into the extracellular medium, so its concentration is a readout of cell density.

The molecular roles of LuxI and LuxR were first elucidated in the marine bacterium *Vibrio fischeri*, where they regulate expression of the *lux* genes responsible for bioluminescence (Fig. S1). The *V. fischeri lux* regulatory region consists of two divergent promoters [5], [6]. At low cell densities, *luxR* is transcribed efficiently from the leftward pL promoter, while *luxI* and bioluminescence genes are transcribed at a basal level from the rightward pR promoter. At high cell densities, AHL-bound LuxR activates transcription at the pR promoter; this initiates a positive-feedback loop via LuxI synthesis. Similar LuxI/LuxR quorum-sensing systems have been identified through sequence homology in over 50 species of gram-negative proteobacteria [7]–[9]. Like *V. fischeri*, many species place LuxI within a positive-feedback loop at the LuxR-regulated promoter (henceforth pR), while LuxR is the target of external regulation [4], [5], [10]–[22] (Table 1). Positive feedback can generate an abrupt switch-like activation of gene expression at some threshold cell density, which can be advantageous in several biological contexts [23], [24]; however, the mere presence of feedback does not guarantee such a response [23]–[27]. Recent experiments on re-wired *V. fischeri* LuxI/LuxR systems have shown that the nature of the response can depend on which protein – LuxI or LuxR – is placed in feedback [28]–[30]. Evidently, a system's actual density-dependent behavior arises from the complex interplay of feedback architecture with microscopic biochemical parameters. However, in order to understand this interplay it seems we must first comprehensively characterize a vast number of relevant parameters – species concentrations, reaction rates, binding constants, and so on. This expanse of biochemical detail presents a fundamental barrier to developing a predictive, experimentally falsifiable description of these systems.

**Table 1:** Examples of feedback and regulation in LuxI/LuxR quorum-sensing systems.

System, function, and feedback architecture		Ref.
*Vibrio fischeri* LuxI/LuxR: Bioluminescence		13,14
Sending	LuxI synthesizes AHL	13
Receiving	AHL binds LuxR, probably drives dimerization	12
Feedback	*luxI* expression activated by LuxR-AHL	13
Regulation	*luxR* expression catabolite-repressed via CRP	5
*Agrobacterium tumefaciens* TraI/TraR: Ti plasmid conjugation		15,16
Sending	TraI synthesizes AHL	17
Receiving	AHL reversibly binds TraR, drives dimerization	17
Feedback	*traI* and *traR* expression activated by TraR-AHL	15
Regulation	*traR* expression octopine-responsive	15
*Pseudomonas aeruginosa* LasI/LasR: Biofilm formation; virulence		18
Sending	LasI synthesizes 3O-C12-HSL	18
Receiving	3O-C12-HSL reversibly binds LuxR, drives multimerization	19
Feedback	*lasI* expression activated by LasR-3O-C12-HSL	20
Regulation	*lasR* expression regulated by a two-component system	4
*Pseudomonas aeruginosa* RhlI/RhlR: Biofilm formation; virulence		18
Sending	RhlI synthesizes C4-HSL	18
Receiving	C4-HSL reversibly binds RhlR homodimer	21
Feedback	*rhlI* expression activated by RhlR-C4-HSL	22
Regulation	*rhlR* expression activated by LasR-3O-C12-HSL	21

Here we show how to cross this biochemical expanse, with the aid of a few carefully chosen measurements. Specifically, we demonstrate that the promoter logic of pR – its transformation of multiple inputs into a single transcriptional output – encapsulates all the biochemical information required to predict the responses of LuxI/LuxR quorum-sensing systems. The idea of summarizing the characteristics of a promoter by its input-output relationship has been a fruitful one in the study of transcriptional networks: gene regulation functions [31], [32], cis-regulatory input functions [33], [34], and genetic logic gates [35], [36] are all variations on this theme. Here we use the term ‘promoter logic function’ to emphasize the fact that the pR promoter integrates multiple regulatory inputs. In contrast to prior usage [36], we do not restrict ourselves to cis-acting inputs alone, but rather take a black-box approach in which the ‘inputs’ can include any upstream elements that influence the output transcription rate. Using a combination of theory and experiment, we show how the promoter logic function of pR is defined and measured; we describe how to predict density-dependent responses from this measurement alone; and we successfully predict the responses of several distinct feedback systems built from *V. fischeri* LuxI/LuxR components. Thus we give concrete meaning to the abstract idea of the promoter as a computational entity, the central processor at the heart of this ubiquitous cell-to-cell communication paradigm.

## Results

### Defining the promoter logic function and density-dependent responses

Consider a thought experiment involving a population of cells whose intracellular LuxI and LuxR concentrations are held fixed (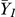,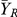). If cell growth is suddenly clamped at some density 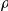, then once sufficient time has elapsed, the AHL concentration (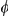) will be proportional to cell density and the LuxI concentration:
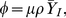
(1)where the proportionality constant 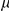 depends on AHL production and decay kinetics, and on the modality of cell growth (Supporting Information, Text S1: Density dependence of AHL). LuxR-AHL binding is in rapid equilibrium [37], so the rate of transcription at the pR promoter will essentially depend on the instantaneous concentrations of LuxR and AHL:
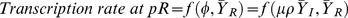
(2)Since we never measure transcription directly, it is convenient define the maximal value of 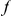 as the unit transcription rate.

The function 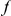 can be interpreted in two distinct but related ways. First, we can consider LuxI and LuxR as its two free inputs, keeping 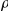 fixed. This is the *promoter logic function* (PLF) of pR, and is valid for feedforward systems in which LuxI and LuxR levels can be set independent of cell density. We can visualize it as the two-dimensional surface generated by varying LuxI and LuxR in the *x* and *y* directions, while plotting the transcriptional output as the height along the *z* direction [33]–[36]. To go from the PLF at density 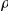 to the PLF at a higher density 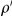, we squeeze the former by the factor 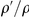 along the LuxI axis; this is equivalent to multiplying the AHL-to-density proportionality constant 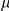 by the same factor.

Alternatively, we can regard 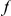 principally as a function of cell density. This interpretation is valid both for feedforward systems with LuxI and LuxR levels held fixed, as well as for feedback systems in which these levels might have density-dependent steady-states 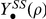. The rate of transcription at pR is then given by:
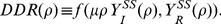
(3)This is the system's *density-dependent response* (DDR); it is visualized as a curve that specifies the transcriptional output at each cell density. Although defined in growth-clamped conditions, the DDR has a clear operational interpretation for growing populations: it is the moving target towards which the transcription rate converges as cell density increases. The more rapidly intracellular components equilibrate relative to cell growth, the closer the actual rate of transcription will be to this target.

**Figure 1:**
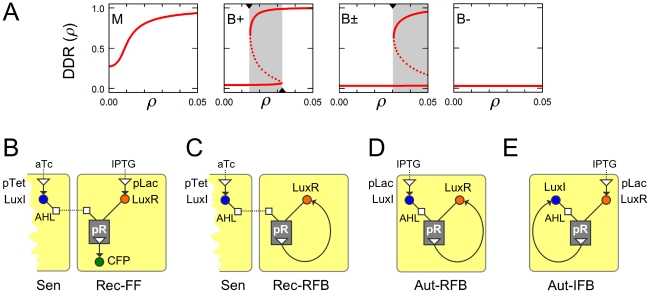
Density-dependent responses and feedback loops. (A) The response of a quorum-sensing system is encapsulated by its transcriptional output, from the moment of inoculation upto its terminal density 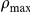. Four different types of density-dependent responses can arise: (M) monostable, where transcription smoothly increases with cell density; (B+) bistable, with a threshold density at which transcription abruptly increases; (B±) bistable and hysteretic at the terminal density, where high and low transcription states co-exist; (B−) bistable but un-induced even at the terminal density, since the potentially bistable region is never reached. Solid lines are stable fixed points, dotted lines are unstable fixed points, and grey boxes indicate bistable density ranges. In our experiments we infer DDRs from the measured terminal responses. These figures were generated for the autonomous LuxI-feedback system using Eq. S18 and parameters from Table S3. Here 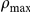 = 0.05 (OD_600_) to match the autonomous loop experiments, while 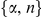 are varied as follows. M: {0.1,0.6}; B+: {0.04,1.6}; B±: {0.01,1.4}; B−: {0.002,1.5}. (B) Constructs used in this study. In sender cells (Sen), LuxI is expressed from the aTc-inducible pTet promoter. In feedforward receiver cells (Rec-FF), LuxR is expressed from the IPTG-inducible pLac promoter, and CFP is expressed from the pR promoter. (C) In the feedback receiver cells (Rec-RFB), LuxR is expressed in feedback from the pR promoter. (D,E) In autonomous feedback systems (Aut-RFB and Aut-IFB) either LuxR or LuxI is expressed in feedback from the pR promoter, while the other protein is expressed from the pLac promoter. Detailed construct maps are given in Tables S1, S2.

We can classify the DDRs of different systems according to their behavior over the relevant cell density range, from zero upto some terminal value 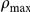 (Fig. 1A; Supporting Information, Text S1: Bifurcation analysis of feedback loops). For monostable DDRs (type M; mnemonic sMooth) transcription is a smoothly increasing, typically sigmoidal function of cell density. For bistable DDRs (type B; mnemonic aBrupt) the sigmoidal curve folds back on itself, so there is a range of cell densities over which two stable transcription levels co-exist. If 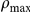 falls beyond the bistable range (type B+), cells that are initially un-induced will abruptly switch to the induced state once their density crosses the threshold at which the lower branch of the curve vanishes. If 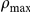 falls within the bistable range (type B±), the system will be hysteretic (history-dependent): cells that are initially un-induced will tend to remain so; cells that are initially induced can sustain induction at the terminal cell density; and noise-driven transitions between these states can generate a heterogeneous population [23], [24]. If 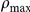 falls below the bistable range (type B−), cells will always remain un-induced; we do not expect this behavior to be relevant in natural contexts. The DDR of a given LuxI/LuxR system will depend on the values of various biochemical parameters, and on the feedback topology; both LuxR and LuxI positive-feedback systems can display all four DDR types, under different parametric conditions.

### Predicting density-dependent responses from promoter logic

Because the PLF and the DDR are essentially different slices of the same function, it should be possible to obtain one from the other as long as they are measured under the same conditions. Here we make a stronger claim: that knowledge of the PLF for a *feedforward* system allows us to predict the entire DDR of *feedback* systems constructed using the same promoter. A feedforward system is one in which both LuxI and LuxR are expressed constitutively while some output protein *Z* (with concentration 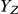) is expressed from pR (Fig. 1B). In a positive-feedback system, either LuxI or LuxR is expressed from pR forming a transcriptional loop, while the other is expressed constitutively (Fig. 1C–E). These possibilities are represented by the following differential equations:
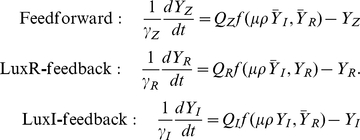
(4)Here, intracellular protein concentrations (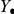) are the dynamical variables; symbols with overbars (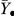) represent the fixed concentrations of constitutively expressed proteins; and the parameters 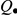 are protein production rates per transcript, scaled by the protein decay rates 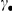. The microscopic biochemical details (hidden inside the function 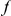) are separable from feedback topology (which determines the structure of the differential equations) – consistent with our intuition that the same genetic components may be re-wired in many ways [29].

**Figure 2:**
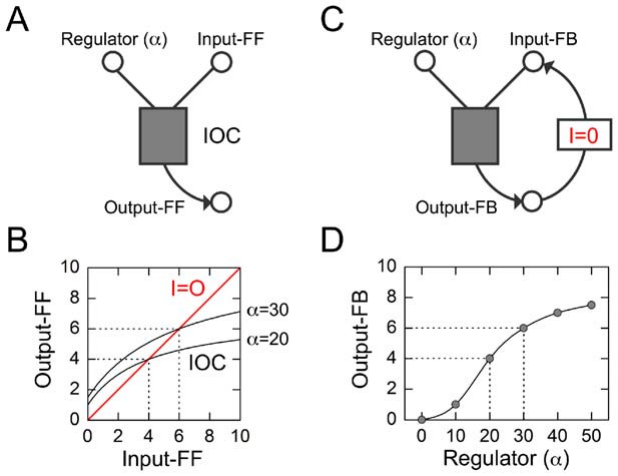
Predicting feedback responses. (A) Consider a black box that transforms a regulatable *input* into a measurable *output*, where properties of this transformation might depend on some external *regulator*. (B) For a fixed regulator value *α*, we map out the *input-output characteristic* (IOC) by varying the input and measuring the resulting output (black curve). (C) If the output is now fed back into the input, the two values are forced to match. This condition only obtains at special points where the IOC intersects the *line of equivalence* I = 0 (red line, Fig. 2B). These intersection points determine all possible steady-state responses of the feedback system, though this graphical argument is agnostic regarding the stability of steady-states. (D) If the regulator level α is now changed, the IOC must again be measured, and the new feedback response predicted. By iterating this process, we obtain the full feedback response as a function of *α*.

Formally, the density-dependent response in the growth-clamped thought experiment can be found by measuring production and decay rates in Eq. 4; the steady-states 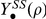 are those protein levels at which these rates become equal [32]. In practice, protein concentrations can be more accurately determined than production and decay rates. A robust, model-independent technique proposed by Angeli *et al.* [26] allows us to predict feedback responses using concentration measurements alone, absent any rate data (Fig. 2). Consider a LuxR-feedback system, where the LuxI concentration is held fixed at a level 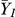 (the *regulator*) and the LuxR concentration is allowed to reach its density-dependent steady-state level 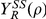 (the quantity we wish to predict). Imagine breaking the feedback loop by expressing LuxR exogenously at its original steady-state level from a constitutive promoter (the *input*), and substituting some passive reporter *Z* in place of LuxR, downstream of pR (the *output*). The concentration of the reporter will be different from that of LuxR because it has a different translation rate: 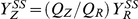. This concentration can also be calculated from Eq. 4, as the system is now identical to the feedforward case: 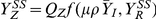. Setting these equal to one another, we see that the steady-state level of LuxR in feedback satisfies a consistency condition:
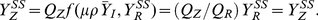
(5)Both the left-hand and right-hand terms can be measured and graphed on a 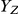 versus 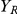 plot (Fig. 2B). The left-hand term is a slice of the PLF; it will generally be a monotonically increasing nonlinear curve called the *input-output characteristic*. The right-hand term will be a straight line called the *line of equivalence* whose slope encodes the input-to-output scale factor. The point 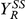 where they intersect satisfies the desired steady-state condition of Eq. 4: it is level LuxR would reach in feedback when the regulator LuxI is held at the given level. A different level 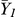 of the regulator corresponds to a different slice of the PLF, and results in a different steady state response 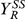 (Fig. 2C,D). The converse of this strategy applies for the LuxI-feedback case: here, the steady-state LuxI response (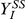) can be predicted as a function of the LuxR regulator level (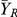). Thus we can predict the response of LuxR or LuxI feedback loops directly from measured PLF at density 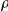; responses at other densities can be predicted using stretched or squeezed versions of the PLF, via the proportionality constant 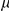.

### From thought experiment to practical measurement

To implement this predictive approach, we must measure the promoter logic in conditions that mimic the idealized thought experiment. Specifically, we must clamp cell growth and AHL accumulation so the AHL-to-density proportionality shown in Eq. 1 is achieved. A continuous-flow chemostat setup clamps cell density rather than cell growth, and is difficult to multiplex. A more feasible strategy relies on the observation that the required AHL-to-density proportionality condition can arise in two very different situations. First: under the static conditions of the thought experiment where cell growth is clamped at a nominal density 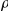. Second: in an exponentially growing culture where LuxI is held constant, and 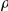 is the cell density *at the time of measurement*. (The only caveat is that the proportionality constant 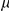 will be different for the two protocols; see Supporting Information, Text S1: Density dependence of AHL.) Under exponential growth conditions, the PLF can be determined by splitting the measurement over two cell types (Fig. 1B): AHL-producing sender cells (Sen) which express LuxI at a pre-determined level; and AHL-responsive receiver cells (Rec-FF) which express LuxR at a pre-determined level, as well as a reporter protein downstream of pR in the feedforward configuration. We first let sender cells grow exponentially from a very low initial density. Once the culture reaches the desired density 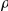, we filter these cells out to clamp AHL levels (which, crucially, now obey the AHL-to-density proportionality condition). Finally, we measure the response of the receiver cells in the filtrate medium. By repeating this measurement at a standard cell density 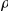 but many different combinations of LuxI and LuxR levels, we can map out the complete PLF.

### The promoter logic function of pR

**Figure 3:**
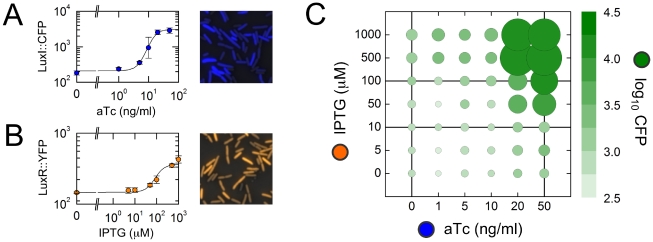
The promoter logic function of pR. (A,B) Input LuxI::CFP and LuxR::YFP values as functions of the inducers aTc and IPTG, respectively; data are fit to Hill functions (Eq. S4) with parameters given in Table S4. To the right of each graph we show inverted phase-contrast images of *E. coli* cells overlaid with pseudocolor fluorescence data of LuxI::CFP and LuxR::YFP levels. Datapoints on the graph are population averaged values of fluorescence-per-pixel in the CFP and YFP channels; error bars represent standard deviations over replicates. (C) Bubble-plot of the measured promoter logic function of pR at the nominal cell density OD_600_ = 0.1. Here, aTc (hence LuxI::CFP) is varied along the *x*-axis; IPTG (hence LuxR::YFP) is varied along the *y*-axis; the area of the circle at each combination of input values represents the resulting CFP output level (which can also be read out using the colorbar). Vertical or horizontal cuts correspond to the input-output characteristics shown in Fig. 5A,D; these can be used to predict LuxR-feedback or LuxI-feedback responses, respectively.

We employed our theoretical framework to predict and test the responses of synthetic quorum-sensing systems built from *V. fischeri* components, expressed in an *Escherichia coli* background [29], [30], [38], [39] (Tables S1, S2). We expressed LuxI in sender cells from the anhydrotetracycline (aTc) inducible *tet* promoter (pTet), and LuxR in receiver cells from the isopropyl β-D-1-thiogalactopyranoside (IPTG)-inducible *lac* promoter (pLac) (Fig. 1B). We grew sender cells in minimal medium containing aTc for 12 h, until the optical density of the culture reached the level OD_600_ = 0.2. At this point we filtered out the cells and retained the AHL-enriched broth, to which we added an equal volume of fresh minimal medium containing IPTG, so that the nominal cell density was OD_600_ = 0.1. We inoculated receiver cells into this broth at low density, and grew them for 12 h to a final density of OD_600_ = 0.1. We tracked the two inputs by measuring the levels of LuxI in sender cells using polycistronic cyan fluorescent protein (designated LuxI::CFP, with fluorescence signal 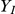) and LuxR in receiver cells using polycistronic yellow fluorescent protein (designated LuxR::YFP, with fluorescence signal 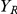). The role of the passive output *Z* was played by the cyan fluorescent protein expressed from pR in receiver cells (designated CFP, with fluorescence signal 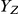). In total we carried out two or more replicate measurements of output CFP for all 42 combinations of 6 different LuxI::CFP levels (varying aTc in the range 0–50 ng/ml; Fig. 3A) and 7 different LuxR::YFP levels (varying IPTG in the range 0–1000 µM; Fig. 3B). Fig. 3C shows the result: the PLF of the *V. fischeri* pR promoter. As expected, the system performs an AND-type operation [35], [36] only generating an output when both LuxI and LuxR levels are above threshold, with the output CFP level varying over two orders of magnitude between the low and high states. Horizontal or vertical slices of the PLF are the *input-output characteristics*.

### Lines of equivalence

We expressed CFP, LuxI::CFP, and LuxR::YFP, in turn, downstream of IPTG-inducible pLac, and used an affine fit to determine fluorescence backgrounds and scale factors to account for the differing translation rates and fluorescence units of each (Fig. S2). For example, plotting LuxR::YFP levels on the *x*-axis and CFP levels on the *y*-axis as IPTG is varied produces a linear fit with non-zero intercept; the background fluorescence levels in each channel can be estimated from intercepts, and the scale factor from the slope (see Supporting Information, Text S1: Fluorescence backgrounds and scale factors). With backgrounds subtracted, on a standard plot the data will fall on a straight line passing through the origin with slope equal to the scale factor; on a log-log plot they will fall on a straight line with unit slope. These correspond to *lines of equivalence*.

### Feedback response measurements

To test the generality of our approach, we predicted and measured the responses of three distinct feedback loops, each for a set of regulator levels, totaling to 20 different DDRs.

Rec-RFB (Fig. 1C): cells expressing LuxR::YFP downstream of pR, giving a LuxR-feedback topology. These feedback receiver cells must be coupled to AHL-producing sender cells expressing LuxI::CFP downstream of pTet. Here, aTc-induced LuxI::CFP is the *regulator*, LuxR::YFP is the *input*, and CFP is the *output*. We predicted the value of LuxR::YFP in feedback for six values of aTc, and compared this to the measured response at terminal density 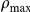 = 0.1 (OD_600_).

Aut-RFB (Fig. 1D): cells expressing LuxI::CFP downstream of pLac, while LuxR is expressed downstream of pR, giving an autonomous LuxR-feedback topology. Here, IPTG-induced LuxI::CFP is the *regulator*, LuxR::YFP is the *input*, and CFP is the *output*. We predicted the value of LuxR::YFP in feedback for seven values of IPTG, and compared this to the measured response at terminal density 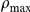 = 0.05 (OD_600_).

Aut-IFB (Fig. 1E): cells expressing LuxR::YFP downstream of pLac, while LuxI is expressed downstream of pR, giving an autonomous LuxI-feedback topology. Here, IPTG-induced LuxR::YFP is the *regulator*, LuxI::CFP is the *input*, and CFP is the *output*. We predicted the value of LuxI::CFP in feedback for seven values of IPTG, and compared this to the measured response at terminal density 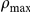 = 0.05 (OD_600_).

In order to detect hysteresis in these experiments, we initialized cells in either un-induced (OFF history) or fully induced (ON history) states before growing them to the terminal density (Materials and Methods: Cell growth and imaging). If the system is hysteretic, the terminal responses of the OFF-history and ON-history cell populations will be different; conversely, if these two populations have similar terminal responses, the system is non-hysteretic. We can infer the DDR type for each feedback construct and regulator level from the measured terminal response alone. If the terminal response is hysteretic, with high and low states, we can immediately conclude that the entire DDR is bistable type B±. If the terminal response is either high or low, but small changes in the regulator level lead to hysteretic behavior, we can infer that the entire DDR is bistable type B+ or B− respectively. If the terminal response is non-hysteretic and ranges over intermediate transcription levels as the regulator level is varied, we can infer that the entire DDR is monostable type M. Ambiguous cases (for example a high terminal response, insensitive to the regulator, is consistent with both types M as well as B+) can be resolved by direct measurement of the DDR over the full density range.

**Figure 4:**
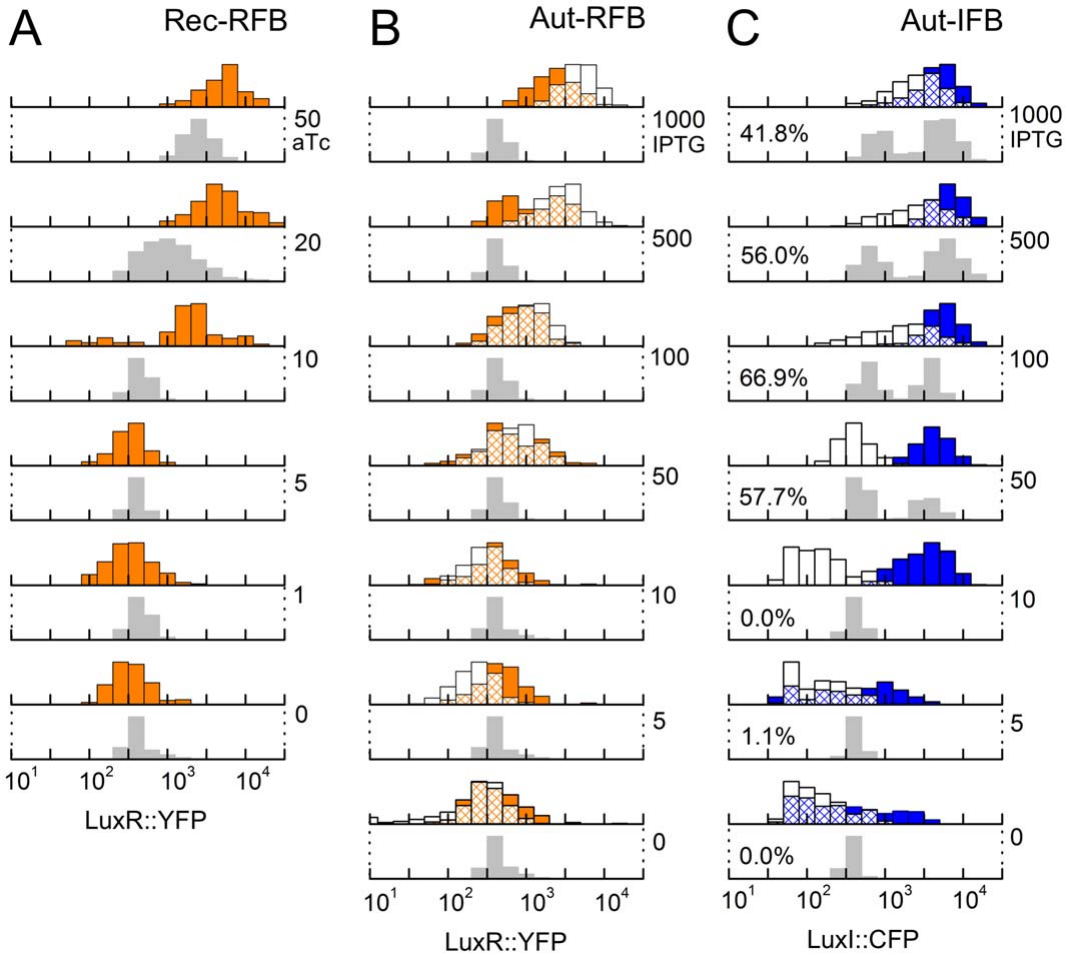
Model-independent predictions. Each stack of histograms relates to predictions and terminal response measurements of a different feedback loop shown in Fig. 1C–E. In all stacks, grey histograms show model-independent predictions over 1000 trials. Note that the predictions are of deterministic steady-states, while the measurements include the effects of cell-to-cell variability; measured histograms are thus broader than predicted ones. (A) Rec-RFB. Orange histograms show observed LuxR::YFP levels. Numbers on the right indicate aTc levels in ng/ml. Our predictions match the observed fluorescence intensities as well as the threshold aTc level within a factor of two, even though both the input and output are varied by over an order of magnitude. (B) Aut-RFB. Orange histograms show observed LuxR::YFP levels for ON-history cells, white histograms show observed LuxR::YFP levels for OFF-history cells; the intersection is hatched. Numbers on the right indicate IPTG levels in µM. LuxR::YFP levels are predicted to be low independent of IPTG, but are observed to be induced starting from IPTG∼50 µM. There is no evidence of hysteresis. (C) Aut-IFB. Blue histograms show observed LuxI::CFP levels for ON-history cells, white histograms show observed LuxI::CFP levels for OFF-history cells; the intersection is hatched. Numbers on the right indicate IPTG levels in µM. For the Aut-IFB case, we sometimes detect three intersections of the input-output characteristic with the line of equivalence, an indication of multistability and hysteresis; the low and high intersections are predicted stable values, the middle intersection is an unstable threshold (e.g. see Fig. 5D). Percent values show the fraction of trials that generate such multistable predictions. The actual terminal response is indeed observed to be hysteretic: histograms from OFF-history cells and ON-history cells are non-overlapping for IPTG = 10 µM and 50 µM.

**Figure 5:**
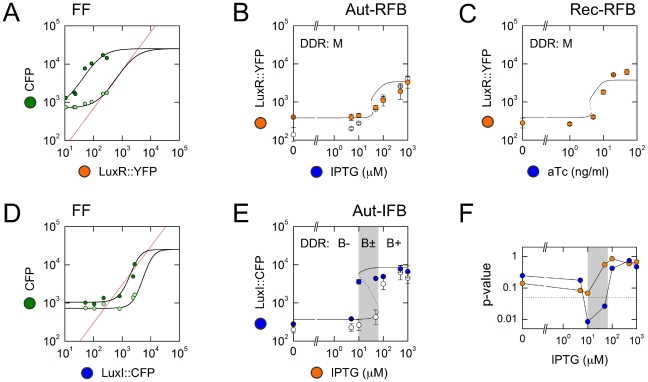
Model-based predictions. (A,D) Intersections of input-output characteristics (IOCs: black curves, generated using Eq. 6, Table S3, and Eq. S4, Table S4) with lines of equivalence (red lines, generated using Eq. S3, Table S3). Datapoints show CFP values from the PLF; fluorescence values are background-subtracted. Since the promoters driving the regulators have lower maximal transcription rates than pR, datapoints lie in a low band of regulator values. Fitted IOCs appear to have the same maximal value because the half-saturation concentration for LuxR-DNA binding is ∼1 LuxR molecule per cell, far below available total LuxR (Supporting Information, Text S1: AHL and LuxR biochemistry). (B,C,E) Predicted (curves) and measured (datapoints) terminal responses for the three feedback loops. Each datapoint gives the mean fluorescence of a cell population; error bars represent standard deviations over replicates. (A) For predicting LuxR-feedback response, the IOC is a vertical slice of the PLF (keeping aTc and LuxI fixed, while varying IPTG and LuxR); for example, we show IOCs corresponding to aTc = 0 ng/ml and 50 ng/ml (Fig. 3C). (B) Feedback response of Aut-RFB. White datapoints show the terminal response of OFF-history cells; orange datapoints show the terminal response of ON-history cells. There is no evidence of hysteresis; we infer that all DDRs are monostable, type M. (C) Feedback response of Rec-RFB. Orange datapoints show measured terminal responses. We infer that all DDRs are monostable, type M. (D) For predicting LuxI-feedback response, the IOC is a horizontal slice of the PLF (keeping IPTG and LuxR fixed, while varying aTc and LuxI); for example, we show IOCs corresponding to IPTG = 10 µM and 100 µM (Fig. 3C). (E) Feedback response of Aut-IFB. White datapoints show the terminal response of OFF-history cells; blue datapoints show the terminal response of ON-history cells; the grey box highlights the hysteretic region. We infer that DDRs in the hysteretic IPTG range are bistable, type B±, while those below and above this range are type B− and B+ respectively. (F) Quantifying hysteresis for autonomous feedback loops Aut-RFB (orange) and Aut-IFB (blue). We show p-values from a T-test quantifying the differences between the terminal responses of ON-history and OFF-history cells over replicates; the dotted line shows p = 0.05. Only the Aut-IFB system shows significant hysteresis (grey box).

Briefly, we find that the Rec-RFB system generates monotonic type M DDRs with no evidence of hysteresis, for all six values of aTc (Figs. 4A, 5C). Similarly, the Aut-RFB system generates non-hysteretic, monotonic type M DDRs for all seven values of IPTG (Figs. 4B, 5B). In stark contrast, the Aut-IFB system generates hysteretic type B± DDRs for a range of intermediate IPTG levels, with un-induced type B− or fully-induced type B+ responses below or above this range (Figs. 4C, 5E). In the following two sections we assess in detail the extent to which our predictions match these observed responses.

### Model-independent predictions

In principle, the prediction procedure is straightforward: holding the regulator fixed, we must extract the appropriate input-output characteristic from the PLF (Fig. 3C), and find its points of intersection with the appropriate line of equivalence (Fig. S2). In practice, there are two complications. First, because the PLF is determined only for a discrete set of input values, some type of interpolation procedure is required before we can detect intersections. Second, two experiments performed with the same construct under different growth conditions will be characterized by different values of the AHL-to-density proportionality constant 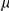, so their DDRs will be relatively stretched or squeezed along the density axis.

As a first pass we opted for a predictive procedure that required no underlying mechanistic model or adjustable parameters: we used power-law interpolation, corresponding to linear interpolation in log-log space [40], and did not allow for any variation in 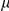. The PLF measurements showed slight deviations between replicates; we incorporated this uncertainty into our prediction procedure using a Monte Carlo approach. Essentially, we added log-normal noise to the measured datapoints and generated an ensemble of predicted intersections over 1000 trials (Materials and Methods: Model-independent predictions). For the Rec-RFB system our model-independent predictions correctly captured, within a factor of two, both the terminal magnitude of LuxR::YFP levels in feedback, as well as the threshold aTc concentration at which the system becomes activated (Fig. 4A). For the Aut-RFB system, although we predicted a consistently low terminal level of LuxR::YFP independent of IPTG concentration, the system was observed to be induced by an order of magnitude starting around IPTG = 50 µM (Fig. 4B). The Aut-IFB case was the most interesting: in this case, we detected multiple intersections of the input-output characteristic with the line of equivalence, implying that the feedback system should be bistable and hysteretic [26]. Indeed, the terminal feedback response showed a strong hysteresis of LuxI::CFP levels (Fig. 4C), and our predictions correctly captured the magnitude of the low and high states. However, we only predicted hysteresis for IPTG≥50 µM, whereas it was observed even at IPTG = 10 µM. Surprisingly, both the autonomous systems were induced *below* the predicted threshold IPTG level, even though they were grown to a lower final density than the receiver cells. We attribute this to the increased accumulation of AHL in the autonomous case, compared with the sender-receiver experiments in which AHL levels decay once sender cells are removed (see Materials and Methods: AHL calibration; Fig. S4).

### Model-based predictions

The variations in the terminal density and AHL levels between different experimental modalities can be captured via the proportionality constant 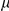. Incorporating this free parameter into the model-independent strategy is cumbersome. To proceed, we employed a more sophisticated interpolation strategy in which we fit the observed PLF to a parameterized functional form. Any function that reasonably described the PLF data would suit our purpose; for example, rational polynomial approximations are frequently used for non-linear systems identification in control theory. A biochemically-motivated form is particularly useful because it can be applied both to predict the macroscopic response of feedback loops, as well as to estimate the values of meaningful microscopic parameters [33], [41]. We used a biochemical model to derive the following parameterized form for the PLF, describing the output CFP level (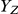) as a function of the two regulated inputs LuxI::CFP (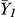) and LuxR::YFP (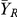) (see Supporting Information, Text S1: Modeling the promoter logic function):
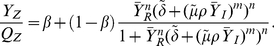
(6)The Hill coefficients *m* and *n* capture the cooperativity of AHL-LuxR binding, and LuxR-DNA binding, respectively; the parameter 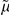 is a scaled version of the AHL-to-density proportionality constant from Eq. 1. We fit this function to the measured PLF, using a non-linear least-squares approach to estimate parameter values [42] (see Supporting Information, Text S1: Parameter estimation; Table S3; Fig. S3). Our estimated Hill coefficients were in reasonable agreement with previous biochemical measurements [12] (see Supporting Information, Text S1: AHL and LuxR biochemistry).

To predict feedback responses, we fed fitted PLF parameters into Eq. 6 to obtain our input-output characteristics (Fig. 5A,D), with the regulator values 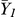 (for LuxR-feedback systems) or 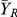 (for LuxI-feedback systems) obtained from Eq. S4. We applied our fitted parameter values directly, with no free parameters or variations in 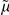, to predict the full set of terminal responses for the Rec-RFB system, for all six values of aTc (Fig. 5C). For the autonomous feedback systems, we kept all PLF parameters fixed save one: the value of 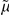 was varied to find the best match between predictions and Aut-IFB data (Table S3: 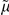
[Aut]). By adjusting this single parameter we were able to predict full set of terminal responses for both the Aut-RFB as well as the Aut-IFB systems, for all seven values of IPTG (Fig. 5B,E).

Our predictions have two substantive components. First, there is the *qualitative* prediction that the Rec-RFB and Aut-RFB systems should be non-hysteretic, and show smooth monotonic DDRs; while the Aut-IFB system should be hysteretic for some regulator levels, and show bistable DDRs. This prediction is robust and model-independent: it relies directly on the measured PLF, with no room for adjustment. That our observations precisely match these qualitative predictions is our strongest result. Second, there is the *quantitative* prediction of the precise induction thresholds and expression magnitudes for each system. These quantitative predictions substantially match the very different observed terminal responses of the Rec-RFB, Aut-RFB and Aut-IFB systems: in all cases we correctly capture the threshold inducer levels and saturating output levels over the full set of regulator values (Fig. 5B,C,E). In two instances, however, the observed response is more gradual than predicted (Fig. 5B,C). This could be due to ‘critical slowing down’ [43], [44], a phenomenon that causes dynamical systems close to a sharp threshold to display slowed kinetics. To explore this further, we used our fitted parameter to predict the *dynamic* responses of the autonomous feedback systems as functions of cell density, under rapid growth conditions. As expected, the observed expression levels lagged behind the dynamic predictions, particularly near sharp thresholds (Fig. S5).

### The interplay of external regulation, promoter logic, and feedback topology

**Figure 6:**
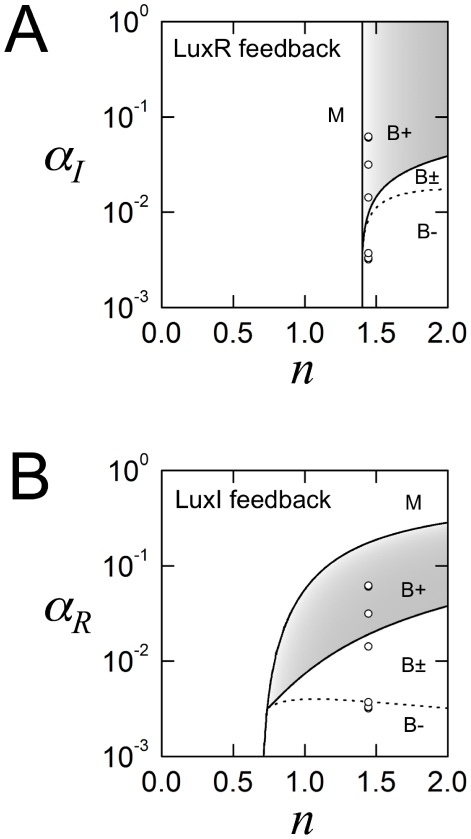
The interplay of regulation, promoter logic, and feedback. (A) Response types for the LuxR-feedback topology, with LuxI as the regulator. (B) Response types for the LuxI-feedback topology, with LuxR as the regulator. Each panel shows an identical slice of parameter space: the Hill coefficient 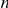 of LuxR-DNA binding is varied along the *x*-axis; the transcription rate 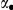 of the regulator is varied along the *y*-axis; all other parameters are fixed at their autonomous loop values given in Table S3. The parameters corresponding to our autonomous loop experiments are shown as seven partly overlapping white dots, whose positions are identical in the two panels: their *x*-coordinates are given by the fitted Hill coefficient 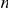 = 1.45; their *y*-coordinates are given by the seven IPTG-induced pLac transcription rates, obtained using Eq. S4 with parameters from Table S4. The boundaries between the four DDR types are computed numerically; any differences in these DDR boundaries between the two panels can be attributed to topology alone. Both LuxR-feedback and LuxI-feedback topologies can generate all four types of density-dependent responses; however, given the same microscopic parameters the two topologies can show distinct behaviors. The observed LuxR-feedback responses happens to fall near the monostable type M boundary, while the observed LuxI-feedback responses are solidly within the bistable type B region. Generically, for a given ‘hard-wired’ value of 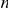 the LuxR-feedback response will be either type M (smooth) or type B (abrupt). In contrast, as long as 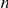 is sufficiently high, the LuxI-feedback system can be tuned between smooth and abrupt responses by varying the regulator level 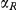. Moreover, the LuxI-feedback system can achieve abrupt responses over a broader range of 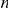 values. These figures are qualitatively unchanged for other values of the fixed parameters (Supporting Information, Text S1: Bifurcation analysis of feedback loops).

The response of a natural LuxI/LuxR feedback system can be modified in three distinct ways: first, the expression level of the regulator could vary, perhaps in response to an external signal; second, the promoter logic function could be perturbed, for example by mutations that influence protein-DNA binding; third, the feedback topology itself could be switched, by large-scale DNA re-arrangements. In our experiments the autonomous LuxR-feedback and LuxI-feedback systems are composed of the same genetic components in permutation (Table S2); this change to the feedback topology, leaving promoter logic untouched, results in systems with completely different qualitative response types. We can use our biochemical model to explore more generally how regulation, promoter logic, and feedback interact to determine system response; Fig. 6 shows our essential findings (Supporting Information, Text S1: Bifurcation analysis of feedback loops; Fig. S6). The two panels, corresponding to the two different feedback topologies, show identical slices of parameter space: the Hill coefficient of LuxR-DNA binding, and therefore the promoter logic function, is varied along the *x*-axis; the expression level of the regulator is varied along the *y*-axis. We see that both feedback topologies can achieve any of the possible response types if parameter values are carefully selected. However, given a set of LuxI/LuxR homologs whose biochemical parameters are randomly assigned, one is much more likely to achieve an abrupt bistable response using a LuxI-feedback topology. Moreover, once biochemical parameters (such as the Hill coefficient) have been fixed, our model predicts that the LuxR-feedback topology is hardwired into a single response type, whereas the LuxI-feedback topology can be tuned between smooth and abrupt responses by varying the LuxR regulator level. This non-trivial prediction is corroborated by the fact that the same LuxI-feedback topology that shows a abrupt bistable response in our experiments generates a smooth monostable response when the LuxR regulator is expressed from a different constitutive promoter [29].

### Dual feedback systems and oscillations

The model-independent strategy we have described here is powerful and broadly applicable: the steady-state response of a feedback system, *if it exists*, will be at one of the self-consistent points where the input-output characteristic intersects the line of equivalence (Fig. 2). However the converse is not true: not all these self-consistent intersections represent feedback steady-states. For example, the LuxI-feedback system has three intersection points (Fig. 5D), but only the low and high intersections represent stable steady-states. It is theoretically possible to predict stability properties if the system under consideration is monotone [26], a subtle technical requirement related to the internal structure of the black box; however, there is no direct method to determine if a given black box is monotone. A feedforward system in which an increase in any input leads to an increase in the output is monotone. From molecular data, we know that the pR promoter with LuxI and LuxR considered as its inputs satisfies this condition. A system with only internal positive feedback loops is also monotone. For example, consider the dual positive feedback case in which LuxI as well as LuxR are expressed downstream of pR (Fig. S7A). One way to decompose this system is to cut the LuxI-feedback loop, and think of the monotone black box as the entire LuxR-feedback system we have already studied (Fig. 5B). The analysis proceeds precisely as before; there is no external regulator, but we can still predict density-dependent responses (Fig. S7B,C; Supporting Information, Text S1: The dual positive-feedback system). However, a black box that contains internal *negative* feedback loops might fail to be monotone. In this situation the model-independent theory is much more difficult to apply [45], but the model-based PLF parameters still prove useful.

**Figure 7:**
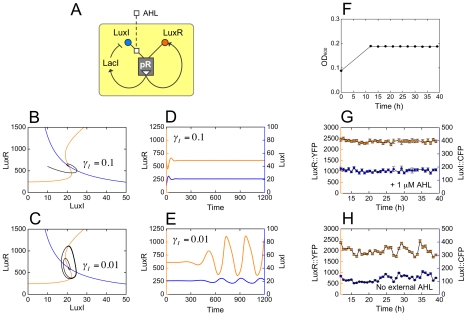
Oscillations in the dual positive/negative-feedback system. (A) In the dual feedback system, both LuxR as well as the LacI repressor are placed downstream of pR. LuxR positively regulates its own expression; LacI negatively regulates the expression of LuxI via the pLac promoter. We model the system using measured PLF parameter values (Table S3), as well as additional parameters describing LacI-pLac interactions and protein decay rates whose values are chosen in order to generate oscillations; the decay rate 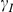 of LuxI is left as a free parameter (Supporting Information, Text S1: The dual positive/negative-feedback system). (B,C) Numerical phase plane analysis. The orange curve is the LuxR nullcline along which 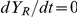; the blue curve is the LuxI nullcline along which 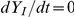; the intersection of these curves is a fixed point which could be stable or unstable. The black curve is shows the system trajectory, which runs counterclockwise as time progresses. (B) For 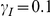 the fixed point is stable, and the system fails to oscillate. (C) For 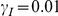 the fixed point is unstable, and the system enters a limit-cycle oscillation. (D,E) We show LuxR (orange, left axis) and LuxI (blue, right axis) values as functions of time (in arbitrary units), corresponding to the trajectories from (B,C). (F) Our experiments are conducted in a nitrogen-limited chemostat, which maintains a steady-state cell density of OD_600_ = 0.185. (G,H) Measured response of dual feedback cells in the chemostat. Datapoints represent the mean fluorescence values of LuxR::YFP (orange, left axis) and LuxI::CFP (blue, right axis) for a population of ∼500 cells; errorbars represent standard error of means. (G) In the control experiment cells are grown in the presence of 1 µM AHL, thus abolishing negative feedback, and the system settles into a steady state. Note that panels (D) and (G) represent very different steady-state situations, and should not be directly compared. (H) In the oscillation experiment cells are initially primed with 1 µM AHL, but this is allowed to dilute out from the 12 h timepoint. From about 20 h, the system displays oscillations that are synchronized over the entire population and stable for 15 h, in qualitative agreement with the numerical predictions.

As a proof-of-principle, we constructed a system involving a dual positive/negative feedback (Fig. 7A); for positive feedback, we used the Aut-RFB system in which LuxR was expressed downstream of pR, while LuxI was expressed downstream of the pLac promoter; for negative feedback, we expressed the repressor LacI downstream of an additional copy of pR, and placed the entire construct in a LacI-deleted *E. coli* strain (Materials and Methods: Dual-feedback experiments). On a LuxR vs. LuxI plot, the self-consistent solution is determined by the intersection of two curves: first, the positive-feedback curve in which the output LuxR is shown as a function of LuxI (Fig. 7B,C: orange curve); second, the negative-feedback curve in which the output LuxI is shown as a function of LuxR, via LacI (Fig. 7B,C: blue curve). The self-consistent solution does exist; however, the PLF parameters on their own cannot be used to determine its stability. To go further, we employed a differential equation formulation containing an extra dynamical parameter: the response rate 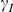 of LuxI (Supporting Information, Text S1: The dual positive/negative-feedback system). For certain values of 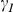 the self-consistent solution is unstable, and the system is predicted to oscillate (Fig. 7C,E). Indeed we observed such oscillations in density-clamped chemostat experiments, synchronized over the entire cell population, with a period of several hours (Fig. 7H). Synchronized oscillations in a LuxI/LuxR-based positive/negative-feedback system have already been reported, and comprehensively analyzed [46]. Our goal here is only to show that, while the PLF on its own does not contain sufficient information to predict oscillations, it nevertheless does restrict the number of additional parameters that need to be considered. Taken together, the success of our predictions demonstrates our central claim: that the promoter logic function of pR contains sufficient biochemical information to determine the feedback responses of diverse quorum-sensing systems.

## Discussion

Any predictive mechanistic description of quorum sensing must be able to connect microscopic rules to macroscopic phenotype. There are essentially three strategies for doing this, distinguished by the level at which measurements must be made. First, we could directly measure all the relevant microscopic biochemical parameters [12]. This approach, while truly predictive, quickly becomes infeasible as the complexity of the system increases and the number of unknown parameters explodes. Second, we could measure the macroscopic response itself, and use these to fit the parameters of a mechanistic model [29]. This approach provides molecular insight and explanatory power, and can be used to rule out models inconsistent with the observed behavior. However, explaining the macroscopic response is not equivalent to predicting it *a priori*. Third, we could make measurements at an intermediate mesoscopic scale, far removed from molecular detail but still below the level of the phenotype of interest. For gene-regulatory networks, this amounts in practice to characterizing the behavior of isolated components of larger feedback systems. Such measurements can be used to estimate ‘lower level’ molecular parameters [33], [41]; but they can also be directly applied to predict ‘higher level’ phenotype [32], [47]. This mesoscopic approach is the one we have taken here. Our central finding is that promoter logic acts as a biochemical focal point: many types of microscopic rules might result in the same promoter logic function, but it is this function alone that determines the macroscopic density-dependent response. This assertion is demonstrated by our ability to predict the density dependent responses of three distinct feedback loops based only on the measured promoter logic, using no free parameters except the density scale. We expect our approach to be broadly applicable, though it will tend to fail if measured promoter logic is perturbed when the system is embedded within a larger network [48], or if the system is influenced by network-host interactions [49].

We have seen that the versatility of LuxI/LuxR quorum-sensing systems arises from the interplay between promoter logic and feedback topology. It is interesting that many transcriptionally characterized LuxI/LuxR systems use a LuxI-feedback configuration, whereas LuxR is typically placed downstream of a promoter that responds to environmental inputs (Table 1). The natural preference for LuxI feedback is unlikely to be the result of a frozen accident, because quorum sensing genes have been repeatedly shuffled over evolutionary timescales [7]. Moreover, this preference cannot be driven by selection for a particular response type, because both topologies can achieve any desired response given the right promoter logic parameter values. We suggest instead that the LuxI-feedback configuration has been selected for its capacity to generate different response types via modulation of the regulator [50]. Feedback topology and promoter logic are ‘hard wired’ (they can only be changed by mutations or re-arrangements at the DNA level), whereas regulator expression can respond dynamically to external cues. This tunability becomes relevant when cells must cope with uncertain or time-varying conditions: the choice between a smooth density-dependent response, abrupt activation, or noise-driven heterogeneity will be dictated by the ecological context. To test this conjecture we would need to observe the quorum-sensing capacities of bacterial species in their natural environment [8], [51], determine whether cell populations do indeed tune their responses, and gauge the extent to which this flexibility has any impact on fitness. As new bacterial genomes are sequenced, the number of putative LuxI/LuxR systems will rise exponentially, and we will be limited only by the rate at which we can experimentally characterize their behavior. The predictive framework we have developed provides a reliable and scalable way to explore the design, function, and diversity of these versatile cell-to-cell communication systems.

## Materials and Methods

### Strains and plasmid constructs

All experiments except those involving the dual-feedback system were performed in the host *Escherichia coli* strain K-12-Z1, a derivative of the K-12 MG1655 strain [52] with a chromosomal gene cassette from the strain DH5αZ1 inserted by P1 transduction [53] (Master's Thesis, S. Dabholkar, 2007). This cassette encodes LacI (the Lac repressor, expressed at ∼3000 copies per cell), TetR (the tetracycline repressor, expressed at ∼7000 copies per cell), and a spectinomycin resistance marker. The inhibition of the pLac promoter by LacI is relieved by the addition of extracellular isopropyl β-D-1-thiogalactopyranoside (IPTG); the inhibition of the pTet promoter by TetR is relieved by the addition of extracellular anhydrotetracycline (aTc). K-12-Z1 cells were maintained at 4°C on LB agar containing 50 µg/ml of spectinomycin; plasmid-transformed cells were maintained on LB agar containing 100 µg/ml of ampicillin. All plasmid constructs were built using components from the Registry of Standard Biological Parts [54] (partsregistry.org). Constructs were assembled using the standard BioBrick assembly strategy, and maintained in the ampicillin-resistant pSB1A2 plasmid backbone (partsregistry.org/Part:pSB1A2) with a pMB1 origin of replication (copy number 100–300). Table S1 lists the BioBrick parts we used; Table S2 gives construct maps.

### Cell growth protocols

Cells from fresh colonies were first grown in 3 ml LB with the appropriate antibiotic for ∼12 h at 37°C. 10 µl of this culture was diluted in 990 µl of 1% glucose-M9 minimal medium [55]. Aliquots ranging from 20–50 µl of this culture were then transferred to several tubes containing 3 ml of 1% glucose-M9 minimal medium with no antibiotic. When required, this medium was supplemented with the appropriate combination of inducers (0, 5, 10, 50, 100, 500, 1000 µM IPTG; 0, 1, 5, 10, 20, 50 ng/ml aTc). These tubes were maintained at 37°C in an incubated shaker for the desired duration, and their final cell density was determined from a 1 ml sample by optical absorbance at 600 nm (OD_600_). The sample closest to the target density was selected for subsequent growth phases when required, or processed for imaging. We used slight variations of this protocol for different constructs:

#### Line of equivalence measurements

Cells transformed with pLac expression constructs (Lac-CFP, Lac-LuxR, or Lac-LuxI; see Table S2) were grown overnight in LB. They were then transferred to 1% glucose-M9 medium containing the desired final concentration of IPTG, allowed to grow for 12 h to a target density OD_600_ = 0.1, and processed for imaging.

#### Sender-receiver measurements

Sender cells (Fig. 1B) were grown overnight in LB. They were then transferred to 1% glucose-M9 medium containing the desired concentration of aTc, and allowed to grow for 12 h to a target density OD_600_ = 0.2. 1 ml of this sample was extracted for imaging. Sender cells were then removed using a 0.22 µm filter (Millipore), and the AHL-containing supernatant was replenished with an equal volume of fresh 2% glucose-M9 medium containing the desired final concentration of IPTG. Receiver cells (Rec-FF or Rec-RFB; Fig. 1B,C) previously grown overnight in LB were added to this medium, grown for 12 h to a final OD_600_∼0.1, then processed for imaging.

#### Autonomous loop hysteresis measurements

ON history protocol: Cells transformed with autonomous feedback loop constructs (Aut-RFB or Aut-IFB; Fig. 1D,E) were grown overnight in LB. They were then transferred to 25 ml of glucose-M9 medium containing 500 nM synthetic AHL and 100 µM IPTG, and grown for 12 h to a target density OD_600_ = 0.2. These cells were washed with 1% glucose-M9 medium by two rounds of centrifugation-pelleting followed by re-suspension, in order to minimize transfer of AHL or IPTG into subsequent steps. OFF history protocol: Cells were grown overnight in LB. They were then transferred to 25 ml of glucose-M9 medium with AHL and IPTG omitted, and grown for 12 h to a target density OD_600_ = 0.05. This lower density was used because OFF-hisotory cells were observed to be partially induced at OD_600_ = 0.2. Both ON and OFF history cells were then transferred, at the appropriate dilution, to 3 ml 1% glucose-M9 medium containing the desired final concentration of IPTG, grown for 12 h to a target density OD_600_ = 0.05, and processed for imaging.

#### Autonomous loop density-dependent measurements

Cells transformed with autonomous feedback loop constructs (Aut-RFB or Aut-IFB; Fig. 1D,E) were grown in 3 ml LB for 8 h. A 5 µl aliquot of this culture was transferred to 80 ml of 1% glucose-M9 medium, and cells were grown for 12 h to a target density OD_600_ = 0.03. These cells were extracted using a 0.22 µm filter, washed twice with glucose-M9 to remove any trace of AHL, and re-suspended in 25 ml of 1% glucose-M9 medium containing the desired concentration of IPTG. Subsequently, at successive timepoints from 0 to 12 h, 1 ml samples were extracted for OD_600_ measurements and then processed for imaging. For the Aut-IFB system, at high cell densities (OD_600_>0.5 after 8+ hours of growth) we found that a sub-population of cells consistently lost fluorescence (possibly due to to plasmid loss, as the media are antibiotic-free; see below). These cells were removed by thresholding on LuxI::CFP levels when calculating population averages. To investigate the cause of fluorescence loss, Aut-IFB cells were imaged at the 0 h and 12 h timepoints; they were then diluted by a factor of 100, and 35 µl of this culture was transferred into 3 ml glucose-M9 medium containing 1 µM AHL. These cells were grown for a subsequent 12 h, then imaged. If bistability were the cause of the low fluorescence population, we would expect fluorescence to recover in the presence of AHL; instead, we see a total fluorescence loss at the 24 h timepoint (Fig. S8A). Colony forming units (CFU) were measured at the 0 h and 12 h timepoints: 50 µl of diluted culture (at dilution factors of 10^4^ or 10^5^) was spread and grown for 12 h at 37°C on LB agar plates containing spectinomycin (25 µg/ml) and ampicillin (50 µg/ml), or spectinomycin alone. Cells were observed to be predominantly ampicillin-resistant at the 0 h timepoint but not at the 12 h timepoint (Fig. S8B), suggesting plasmid loss as the cause of fluorescence loss.

### Microscopy and image analysis

Cells from 1 ml of culture were pelleted by centrifugation at 13.2 k rpm for 10 minutes at 37°C, then re-suspended in 10 µl of 1% glucose-M9 medium. 4 µl of this suspension was placed on a microscope slides (Thomas Scientific), and pressed gently under a coverslip. Samples were imaged on a fully automated Zeiss Axiovert M200 epifluorescence microscope with a cooled CCD camera (Princeton Instruments Pixis). Phase contrast images as well as fluorescence images using CFP and YFP filter sets (Chroma) were acquired for each field of view. 8–15 fields were imaged from a given sample, depending on the cell density. (A small proportion of the imaging was performed on an Olympus IX81 microscope in DIC and fluorescence modes; standard calibration curves were used to match CFP and YFP values between the Olympus and Zeiss instruments. Dual-feedback oscillation experiments were conducted on the Zeiss system with an altered camera gain for improved signal.) Images were analyzed using the MATLAB image processing toolbox (Mathworks). Phase contrast or DIC images were used to generate a binary cell mask, with morphological constraints used to filter out non-cell objects. The mask was then applied to fluorescence images to calculate the average CFP and YFP intensity per pixel of single cells, for a population of ∼500 cells in each sample. Single-cell fluorescence values were approximately log-normally distributed; population-averaged fluorescence signals were calculated as the geometric mean of single-cell values, and error bars were determined as standard deviations between means over replicates.

### Model-independent predictions

We estimated the relevant input-output characteristic using power-law interpolation of the measured PLF [40], then determined its intersection(s) with the relevant line of equivalence to generate predictions. Rec-RFB: On a log-log plot of CFP vs. LuxR::YFP (determined from the PLF by holding aTc, therefore LuxI::CFP, fixed), we connected datapoints by straight lines and enumerated all the intersections with the line of equivalence. Aut-RFB: Here, IPTG is used to modulate the regulator LuxI::CFP, which takes on values distinct from those used to determine the PLF. To account for this, we first generated predicted intersections for each aTc-induced LuxI::CFP level of the PLF, as with Rec-RFB. We then used power-law interpolation to find predicted values at the desired IPTG-induced LuxI::CFP level. Aut-IFB: On a log-log plot of CFP vs. LuxI::CFP (determined from the PLF holding IPTG, therefore LuxR::YFP, fixed), we connected datapoints by straight lines and enumerated all the intersections with the line of equivalence. In all instances, we conservatively extrapolated below and above the domain of measurement using flat lines. By repeating the log-log interpolation and intersection procedure 1000 times with noise added to the datapoints based on standard errors of measurement, we generated a list of predicted intersections.

### AHL calibration

Rec-FF cells were grown in LB for 10 h, transferred to 3 ml glucose-M9 minimal medium prepared with 20 µM of synthetic AHL (Sigma-Aldrich), then grown for varying periods (0, 4, 8, and 12 h) to a target density OD_600_ = 0.1. The culture was centrifuged at 8000 rpm for 20 min at 4°C. AHL from the supernatant was extracted twice using an equal volume of ethyl acetate. Extracted samples were dried using a centrifugal evaporator (Labconco) at 35°C for 30 min. Pellets were resuspeneded in 1 ml solution of MilliQ water (60%) and methanol (40%). Samples were analyzed by HPLC (Shimadzu) using an RP-18e column (Purospher STAR, 250×4.6 mm, 0.5 µM). Components were isocratically eluted with 60∶40 water/methanol (v/v) at a total flow rate of 0.4 ml/min, and the absorption at 253 nm was recorded (Fig. S4A, inset). All solvents and samples in this protocol were acidified with 0.1 ml/l acetic acid, as AHL is unstable at alkaline pH. The same protocol was then repeated omitting the introduction of Rec-FF cells at the first step. We obtained a linear calibration between AHL concentration and peak area (Fig. S4A), and found that the measurement was sensitive down to an AHL concentration of ∼1 µM. Rec-FF cells did not appreciably affect AHL degradation, which occurs with a half-life of 4.3±0.2 h in the absence of cells, and 4.0±0.1 h in their presence (Fig. S4B). Terminal AHL levels in our feedforward and feedback experiments were below the detection limit of the HPLC protocol, and could not be directly measured. We therefore titrated synthetic AHL against the response of Rec-FF cells, with LuxR induced using 500 µM IPTG. Cells were grown in glucose-M9 medium with varying AHL concentrations for 12 h, upto a target density OD_600_ = 0.1, and their CFP expression was determined by imaging. The response curve is best fit with a Hill coefficient of 1.9±0.5, and a half-saturation value of 825 nM AHL (Fig. S4C). This half-saturation value is an over-estimate as AHL decays over the 12 h duration of the experiment; biochemical measurements [12] suggest a value closer to 85 nm (Fig. S4D).

### Dual feedback experiments

The construction of the dual positive/negative-feedback system has been described in Anand et al [48]. Briefly, the Aut-RFB construct was extended by placing LacI downstream of an additional copy of the pR promoter, while LuxI was expressed downstream of a CRP-dependent pLac promoter. This ampicillin-resistant plasmid construct was transformed into the *lacI* deleted kanamycin-resistant *E. coli* strain JW0336-1 (CGSC, Yale University). Cells from fresh colonies were first grown in 3 ml LB for 5 h; a 1 ml aliquot was then diluted to a final volume of 100 ml using 1% succinate-M9 medium containing 1 µM AHL, and grown for 5 h to a density of OD_600_∼0.15. Cells were extracted by centrifugation at 5000 rpm for 4 minutes at 25°C, and re-suspended in 2 ml warm nitrogen-limited (1 mM NH_4_Cl) succinate-M9; 1 ml portions were transferred into two replicate flasks and diluted upto 50 ml with nitrogen-limited succinate-M9 medium containing 1 µM AHL. A nitrogen-limited chemostat culture was established at 37°C at a dilution rate of 0.15/h, operating at a steady-state OD_600_ = 0.185. During first 12 h, the source flask was replenished every 4 h with fresh medium containing 1 µM AHL; this was done to ‘prime’ the system into a high LuxR state. At this 12 h timepoint in the oscillation experiment, AHL-absent medium was provided in the source flask, causing AHL in the growth flask to dilute out. In control experiments, the source flask continued to be replenished every 4 h with AHL-containing medium. All growth media in these experiments contained 100 µg/ml ampicillin. OD_600_ was measured every 3 h, and cells were imaged every 45 min as described above.

## Supporting Information

Figure S1 **LuxI/LuxR quorum-sensing systems.** LuxI (blue circle) is an enzyme that synthesizes acyl-homoserine lactone (AHL; white square). LuxR (orange circle) is a transcriptional activator. (A) At low cell densities, LuxR is expressed at high levels from the pL promoter, while LuxI is expressed at a basal level from the pR promoter. AHL is synthesized at low levels, and diffuses freely across the cell membrane. LuxR remains in an inactive form. (B) At high cell densities, the aggregate synthesis of AHL from many cells drives up its extracellular and intracellular concentration, promoting LuxR-AHL binding. AHL-bound LuxR activates transcription of LuxI at the pR promoter, driving a positive feedback loop.
(TIF)

Figure S2 **Measuring lines of equivalence.** We determined CFP, LuxR::YFP, and LuxI::CFP values for proteins expressed from pLac with IPTG = [0 5 10 50 100 500] µM. Each datapoint gives either the (A) LuxR::YFP or (B) LuxI::CFP level against the corresponding CFP level at equal IPTG concentrations; error bars represent standard errors of measurement over replicates. The lines of equivalence (red) are determined by affine fits.
(TIF)

Figure S3
**Promoter logic parameter estimation.** (A) We estimated the parameters of Eq. 6/Eq. S14 by non-linear least-squares fitting. We observed for an unconstrained fit that the value of the LuxR-AHL binding Hill coefficient 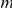 increased without bound; but if the value of 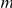 was fixed, the algorithm robustly converged to a set of best-fit parameters. Here we show fitted parameter values as a function of 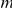. The chi-square error (top left graph) decreases monotonically with 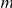; this underlies the numerical instability. Throughout the paper, parameter values are those determined for 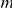 = 2. The value of the LuxR-DNA binding Hill coefficient 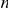 is only weakly dependent on 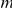 (bottom right graph). (B) Predicted vs. observed CFP values for the 42 datapoints of the PLF, from a 5-parameter fit. (C) The histogram shows the distribution of chi-square values found for 1000 Monte Carlo trials using synthetic datasets. A fraction Q = 0.8 of these values are greater than value from the actual fit (vertical red line), showing that the deviations in Fig. S3B are within measurement error.(TIF)

Figure S4
**AHL calibration.** (A) The area under the curve from HPLC measurements of absorption at λ = 253 nm, plotted against synthetic AHL concentration. The inset shows the absorption peak. (B) AHL decay measured using HPLC. The exponential fit shows that AHL decays with a half-life of ∼4 h, independent of the presence or absence of cells in the medium. (C) Titration: the CFP levels of Rec-FF cells (with LuxR induced using 500 µM IPTG) plotted against the initial levels of synthetic AHL in the medium. The curve shows a Hill fit, with the best fit Hill coefficient 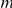 = 1.94±0.5. (D) Data from gel-shift experiments of LuxR-to-AHL binding for 3.5 nm total LuxR, as a function of AHL levels. The curve shows a fit with the Hill coefficient fixed at 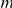 = 1.94. Datapoints estimated graphically from figures in Urbanowski *et al.* [12]. (E) Data from DNA protection experiments probing the binding of LuxR-AHL to DNA as a function of LuxR levels, when AHL is in excess (10 µM). The curve shows a fit with the Hill coefficient fixed at 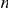 = 1.45, as estimated from our PLF measurements. Datapoints estimated graphically from figures in Urbanowski *et al.* [12].(TIF)

Figure S5
**Dynamic predictions and responses.** We predicted the entire density-dependent response of the two autonomous loop constructs (using Eqs. S17 and S18, with parameters from Table S3), starting from low density and going up to the carrying capacity of our media (OD_600_∼1). As expected given the high rates of change of cell density under these conditions, the observed feedback response lags the predicted DDR at all times. Nevertheless, the predictions correctly capture how changes in the regulator level accelerate the induction dynamics. Grey curves show the DDR predicted from Eqs. S17 and S18, with parameters from Table S4. In principle the parameter 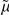 should be re-calculated for these new high-density growth conditions, but we have used 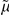 [Sen] directly (Table S3). Datapoints show the observed responses for (A) the Aut-RFB system and (B) the Aut-IFB system. We determined responses at two different IPTG concentrations (hence two different levels of the regulator LuxI::CFP or LuxR::YFP, respectively). Measurements were made at 2 h intervals until the cultures entered stationary phase.(TIF)

Figure S6
**Mapping the boundary between monostable and bistable regions.** Using autonomous loop parameters from Table S3 and a fixed value of cell density 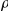, we can find the regions of 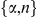 space that admit bistable solutions (regions within the taper emanating from a critical point, bounded by a set of black and red curves). As 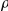 is increased up to the level 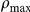, these tapers move toward lower values of 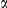. Any given point will transition from the un-induced (below taper) to the bistable (within taper) to the fully induced (above taper) regions, thus mapping out the DDR as a function of 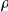. Once we reach 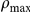, any point above the taper would have already been induced (B+); any point still inside the taper would be hysteretic (B±); and any point below the taper would be un-induced (B−); Fig. 6 was generated for 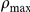 = 0.05. By tracing out the critical points as cell density is increased from 0 to ∞, we can find the line that separates the type M and type B regions.(TIF)

Figure S7
**The dual positive-feedback system.** (A) Dual positive feedback is achieved by placing both LuxR and LuxI downstream of pR. This system has no regulator, but is still sensitive to extracellular AHL levels. (B) We model the system using the autonomous loop parameters from Table S3. We further allow the relative translational efficiencies of LuxI and LuxR to be tuned: the condition 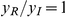 means we use the directly measured translation rates, while 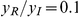 is equivalent to LuxR having a 10-times reduced translation rate (Eq. S27). As in Fig. 1 of the main text, we solve for the density-dependent response (DDR) of the system for various values 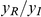, and of the Hill coefficient 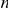. As 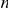 is increased, the system moves from type M (white), through type B+ (grey) and eventually to type B± (white). (C) Sample DDRs, for 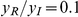, and 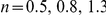 (shown as open circles in panel B).(TIF)

Figure S8 **Fluorescence loss measurements.** (A) We sampled Aut-IFB cells from various timepoints of the density-dependent protocol (see Materials and Methods: Autonomous loop density-dependent measurements). Cells were extracted for imaging, then re-diluted, just prior to the 0 h, 12 h, and 24 h timepoints; the OD_600_ values indicated correspond to pre-dilution densities. Three replicates of the same experiment are shown. Maximal LuxI::CFP fluorescence values increase throughout the first 12 h growth phase; however, a sub-population of cells show loss of fluorescence. Addition of AHL and subsequent growth to the 24 h timepoint does not lead to fluorescence recovery, indicating that the loss is irreversible. (B) Our constructs are carried on an ampicillin-resistant plasmid backbone. We measured the number of colony-forming units (CFUs) per ml of sample from the 0 h and 12 h extracts, in the presence and absence of ampicillin; errorbars represent standard error of the mean over triplicates. At 0 h all cells are ampicillin resistant (no significant difference between the two counts, p = 0.89), while at 12 h the fraction of resistant cells has fallen to less than a fifth (p = 0.003), suggesting plasmid loss is responsible for loss of fluorescence.
(TIF)

Table S1 **List of BioBrick parts.**
(PDF)

Table S2 **Construct maps.**
(PDF)

Table S3 **pR promoter logic parameter values.**
(PDF)

Table S4 **Inducible promoter parameter values.**
(PDF)

Text S1 **Supporting theory, tables, and figures.**
(PDF)
