# Supplementary figures and images for: Prediction by Promoter Logic in Bacterial Quorum Sensing
Source: PLoS Comput Biol. 2012 Jan 19;8(1):e1002361. doi: 10.1371/journal.pcbi.1002361 (PMC3261908; doi:10.1371/journal.pcbi.1002361)

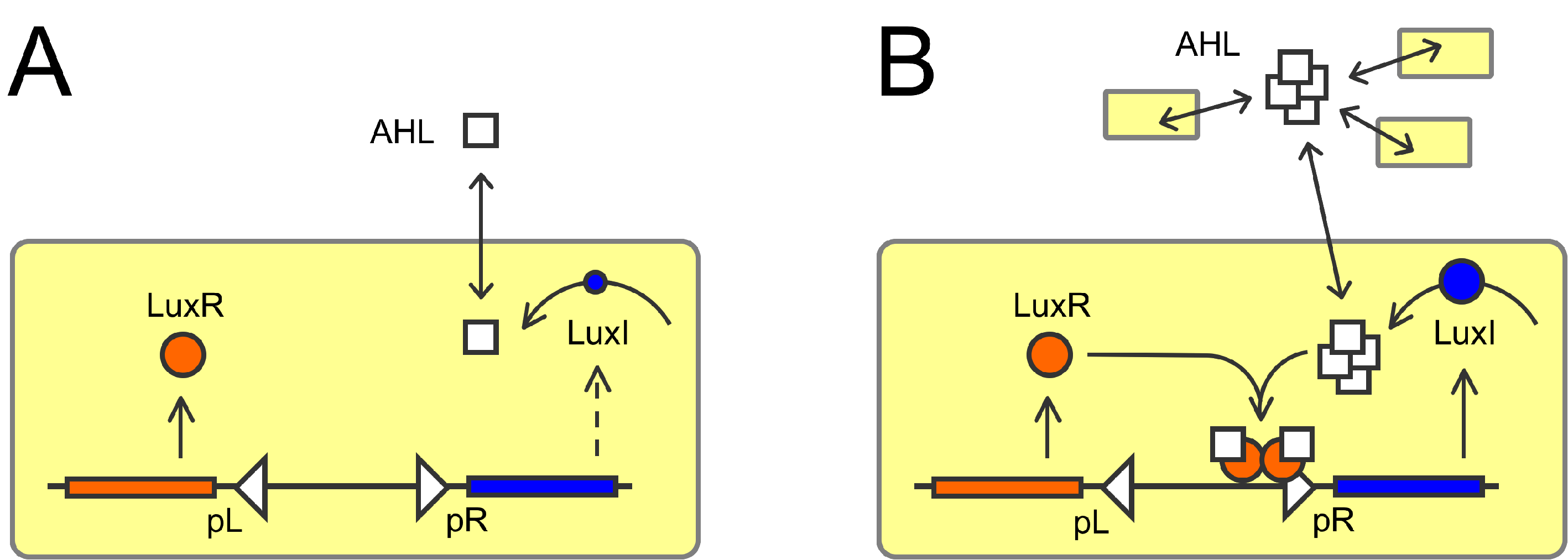

Supplement: Figure S1 — LuxI/LuxR quorum-sensing systems. LuxI (blue circle) is an enzyme that synthesizes acyl-homoserine lactone (AHL; white square). LuxR (orange circle) is a transcriptional activator. (A) At low cell densities, LuxR is expressed at high levels from the pL promoter, while LuxI is expressed at a basal level from the pR promoter. AHL is synthesized at low levels, and diffuses freely across the cell membrane. LuxR remains in an inactive form. (B) At high cell densities, the aggregate synthesis of AHL from many cells drives up its extracellular and intracellular concentration, promoting LuxR-AHL binding. AHL-bound LuxR activates transcription of LuxI at the pR promoter, driving a positive feedback loop. (TIF) [file pcbi.1002361.s001.tif]

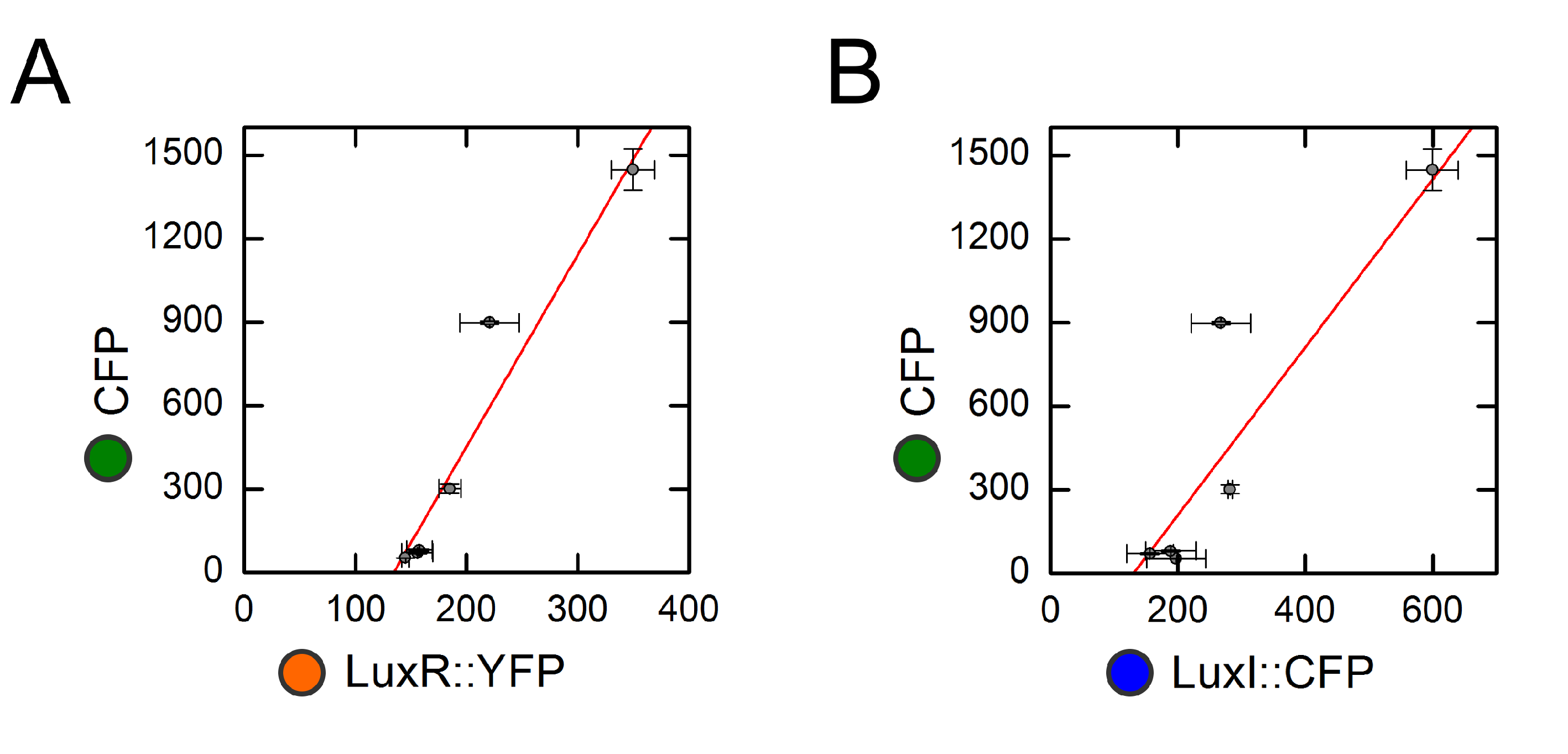

Supplement: Figure S2 — Measuring lines of equivalence. We determined CFP, LuxR::YFP, and LuxI::CFP values for proteins expressed from pLac with IPTG = [0 5 10 50 100 500] µM. Each datapoint gives either the (A) LuxR::YFP or (B) LuxI::CFP level against the corresponding CFP level at equal IPTG concentrations; error bars represent standard errors of measurement over replicates. The lines of equivalence (red) are determined by affine fits. (TIF) [file pcbi.1002361.s002.tif]

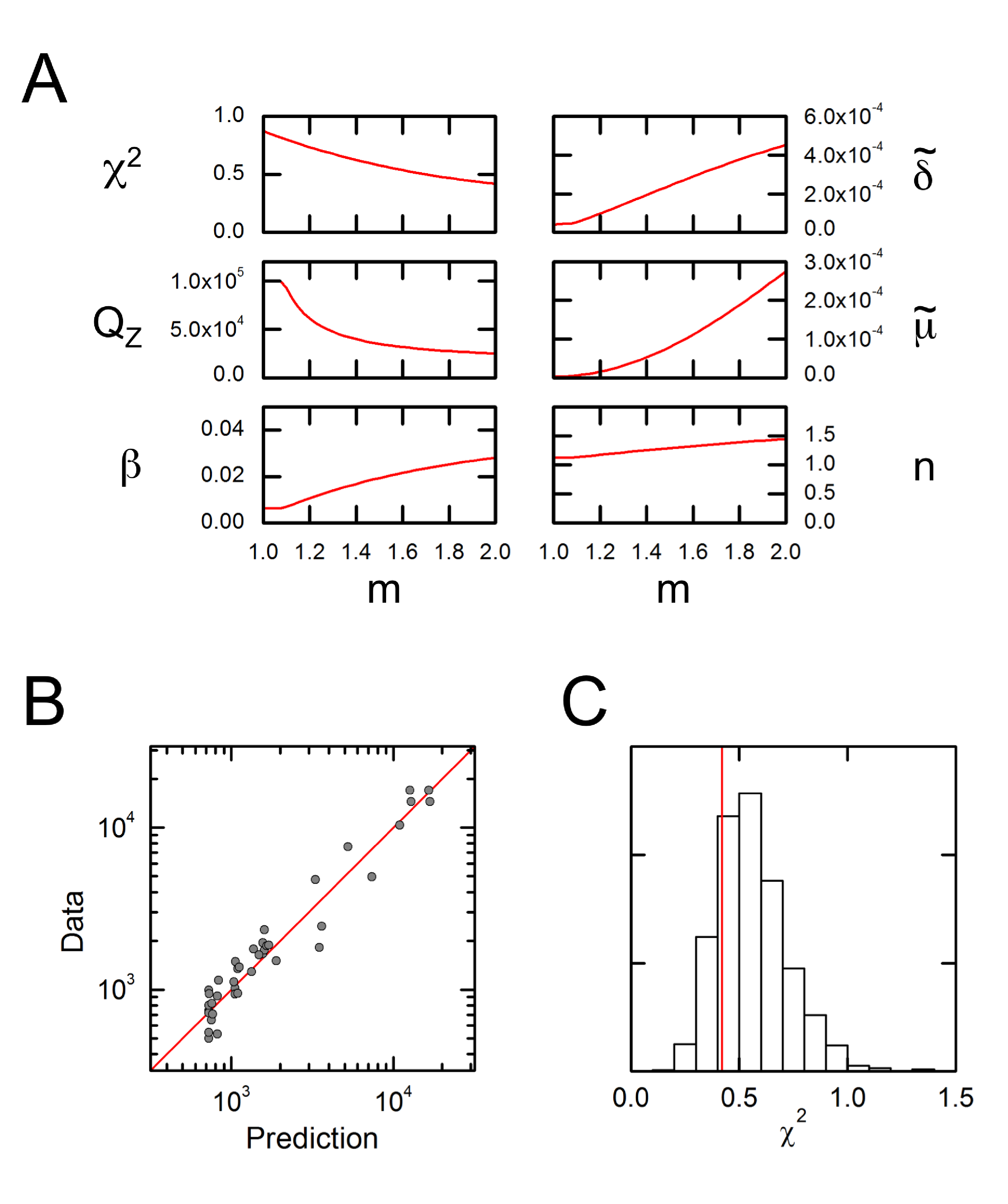

Supplement: Figure S3 — Promoter logic parameter estimation. (A) We estimated the parameters of Eq. 6/Eq. S14 by non-linear least-squares fitting. We observed for an unconstrained fit that the value of the LuxR-AHL binding Hill coefficient increased without bound; but if the value of was fixed, the algorithm robustly converged to a set of best-fit parameters. Here we show fitted parameter values as a function of . The chi-square error (top left graph) decreases monotonically with ; this underlies the numerical instability. Throughout the paper, parameter values are those determined for = 2. The value of the LuxR-DNA binding Hill coefficient is only weakly dependent on (bottom right graph). (B) Predicted vs. observed CFP values for the 42 datapoints of the PLF, from a 5-parameter fit. (C) The histogram shows the distribution of chi-square values found for 1000 Monte Carlo trials using synthetic datasets. A fraction Q = 0.8 of these values are greater than value from the actual fit (vertical red line), showing that the deviations in Fig. S3B are within measurement error. (TIF) [file pcbi.1002361.s003.tif]

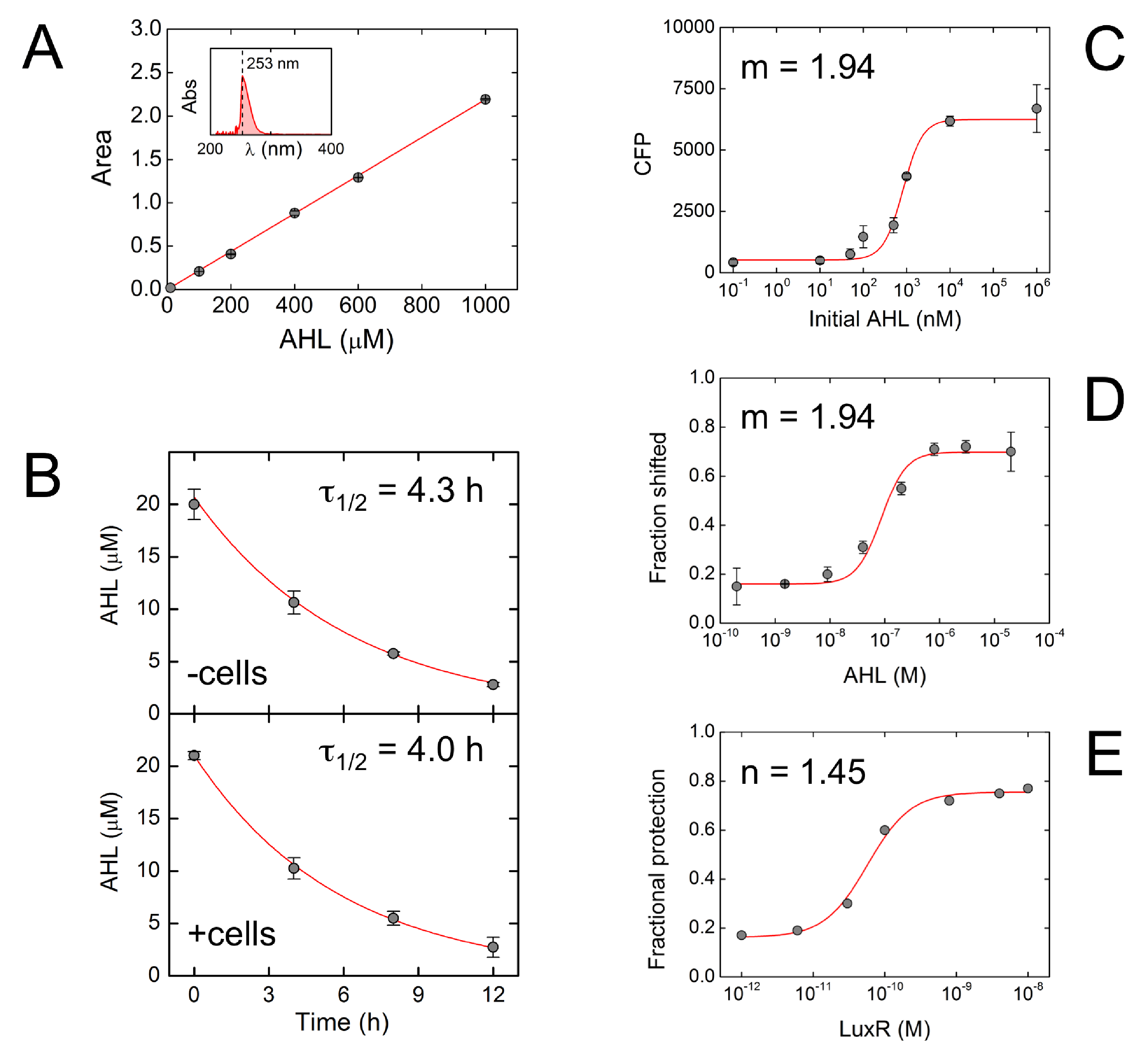

Supplement: Figure S4 — AHL calibration. (A) The area under the curve from HPLC measurements of absorption at λ = 253 nm, plotted against synthetic AHL concentration. The inset shows the absorption peak. (B) AHL decay measured using HPLC. The exponential fit shows that AHL decays with a half-life of ∼4 h, independent of the presence or absence of cells in the medium. (C) Titration: the CFP levels of Rec-FF cells (with LuxR induced using 500 µM IPTG) plotted against the initial levels of synthetic AHL in the medium. The curve shows a Hill fit, with the best fit Hill coefficient = 1.94±0.5. (D) Data from gel-shift experiments of LuxR-to-AHL binding for 3.5 nm total LuxR, as a function of AHL levels. The curve shows a fit with the Hill coefficient fixed at = 1.94. Datapoints estimated graphically from figures in Urbanowski et al. [12]. (E) Data from DNA protection experiments probing the binding of LuxR-AHL to DNA as a function of LuxR levels, when AHL is in excess (10 µM). The curve shows a fit with the Hill coefficient fixed at = 1.45, as estimated from our PLF measurements. Datapoints estimated graphically from figures in Urbanowski et al. [12]. (TIF) [file pcbi.1002361.s004.tif]

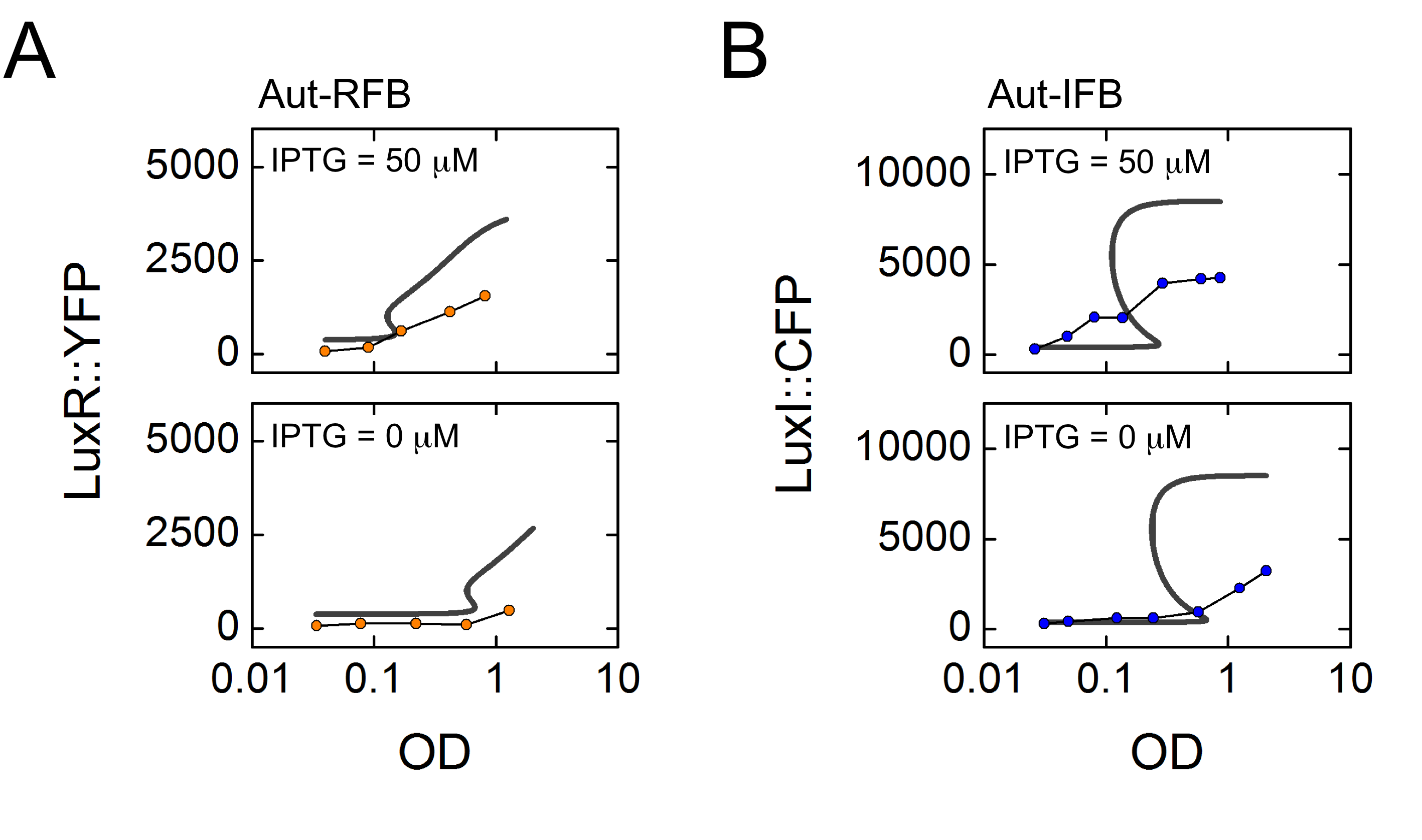

Supplement: Figure S5 — Dynamic predictions and responses. We predicted the entire density-dependent response of the two autonomous loop constructs (using Eqs. S17 and S18, with parameters from Table S3), starting from low density and going up to the carrying capacity of our media (OD600∼1). As expected given the high rates of change of cell density under these conditions, the observed feedback response lags the predicted DDR at all times. Nevertheless, the predictions correctly capture how changes in the regulator level accelerate the induction dynamics. Grey curves show the DDR predicted from Eqs. S17 and S18, with parameters from Table S4. In principle the parameter should be re-calculated for these new high-density growth conditions, but we have used [Sen] directly (Table S3). Datapoints show the observed responses for (A) the Aut-RFB system and (B) the Aut-IFB system. We determined responses at two different IPTG concentrations (hence two different levels of the regulator LuxI::CFP or LuxR::YFP, respectively). Measurements were made at 2 h intervals until the cultures entered stationary phase. (TIF) [file pcbi.1002361.s005.tif]

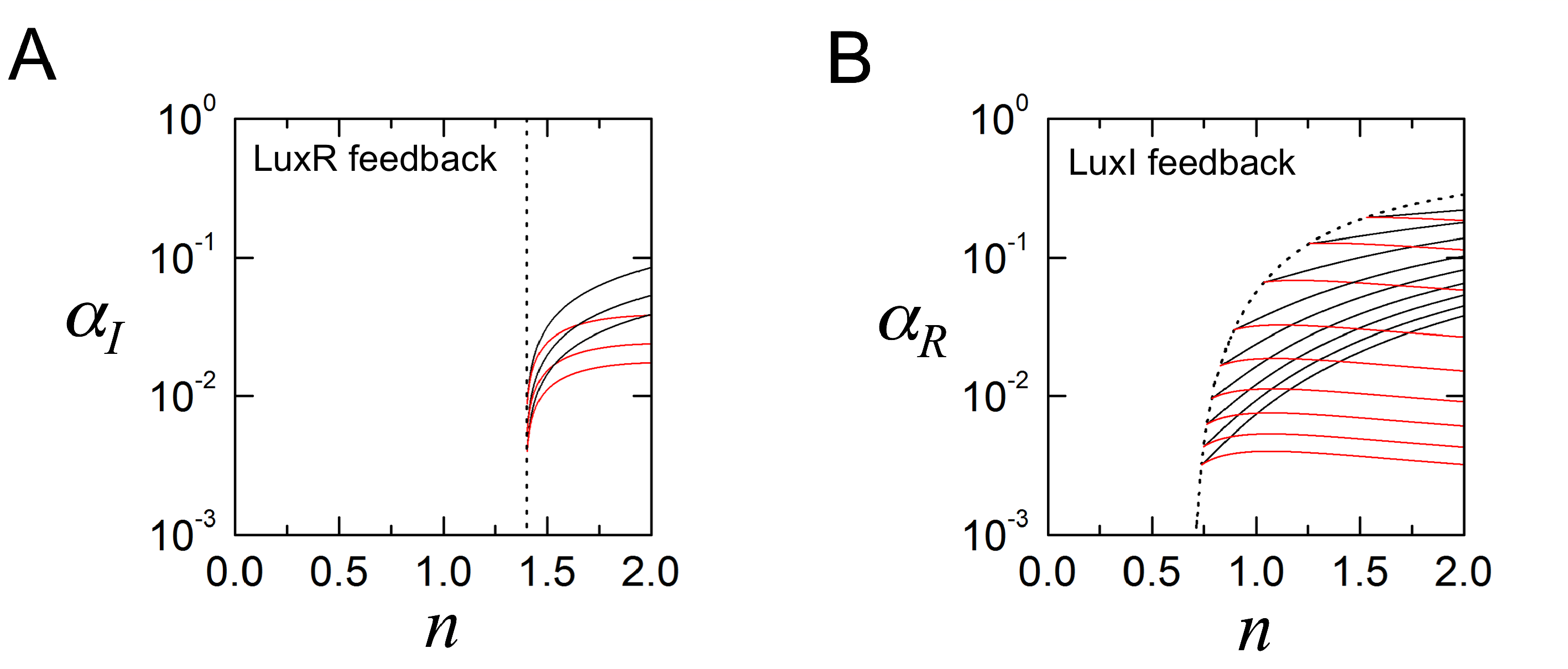

Supplement: Figure S6 — Mapping the boundary between monostable and bistable regions. Using autonomous loop parameters from Table S3 and a fixed value of cell density , we can find the regions of space that admit bistable solutions (regions within the taper emanating from a critical point, bounded by a set of black and red curves). As is increased up to the level , these tapers move toward lower values of . Any given point will transition from the un-induced (below taper) to the bistable (within taper) to the fully induced (above taper) regions, thus mapping out the DDR as a function of . Once we reach , any point above the taper would have already been induced (B+); any point still inside the taper would be hysteretic (B±); and any point below the taper would be un-induced (B−); Fig. 6 was generated for = 0.05. By tracing out the critical points as cell density is increased from 0 to ∞, we can find the line that separates the type M and type B regions. (TIF) [file pcbi.1002361.s006.tif]

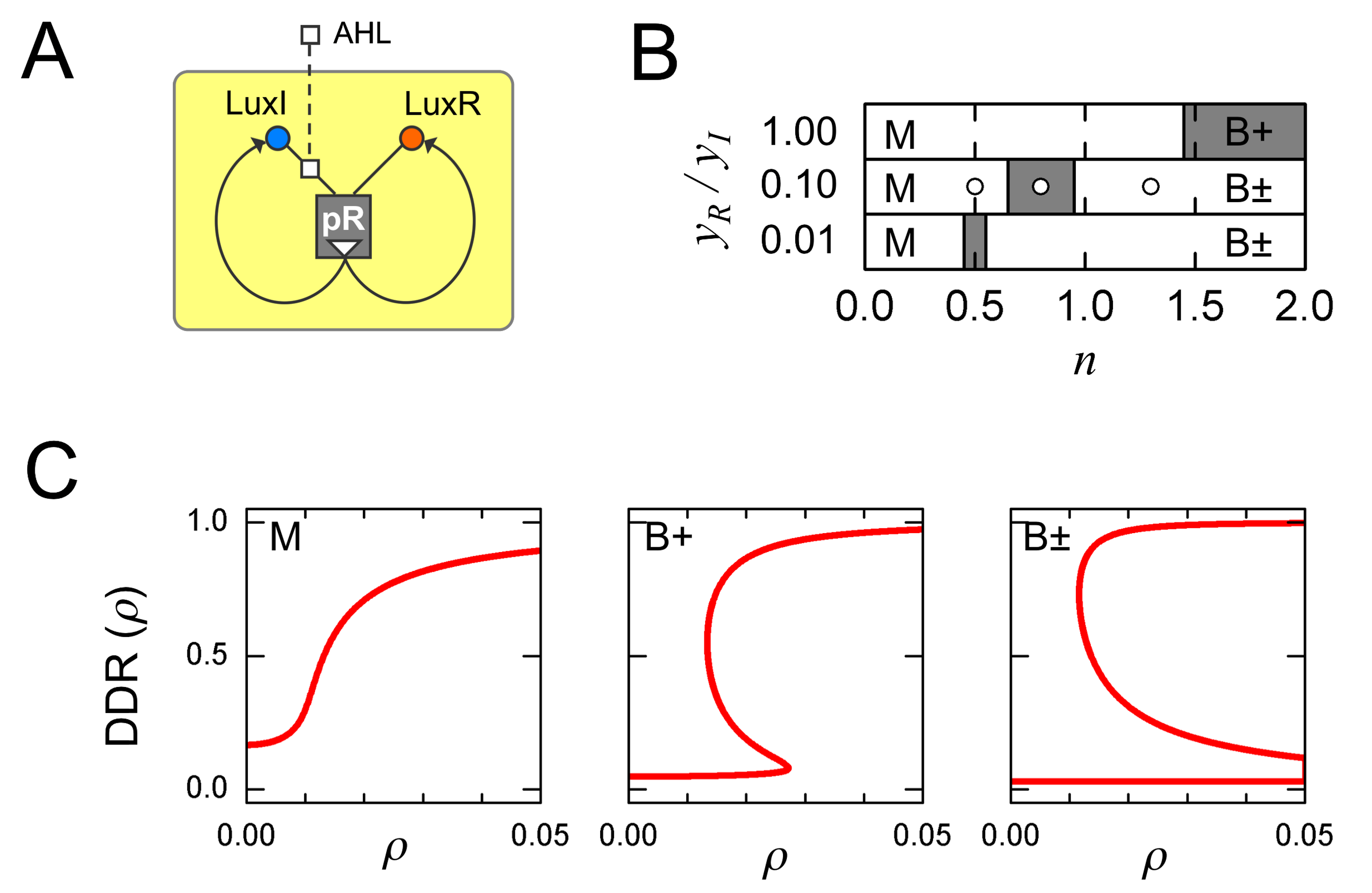

Supplement: Figure S7 — The dual positive-feedback system. (A) Dual positive feedback is achieved by placing both LuxR and LuxI downstream of pR. This system has no regulator, but is still sensitive to extracellular AHL levels. (B) We model the system using the autonomous loop parameters from Table S3. We further allow the relative translational efficiencies of LuxI and LuxR to be tuned: the condition means we use the directly measured translation rates, while is equivalent to LuxR having a 10-times reduced translation rate (Eq. S27). As in Fig. 1 of the main text, we solve for the density-dependent response (DDR) of the system for various values , and of the Hill coefficient . As is increased, the system moves from type M (white), through type B+ (grey) and eventually to type B± (white). (C) Sample DDRs, for , and (shown as open circles in panel B). (TIF) [file pcbi.1002361.s007.tif]

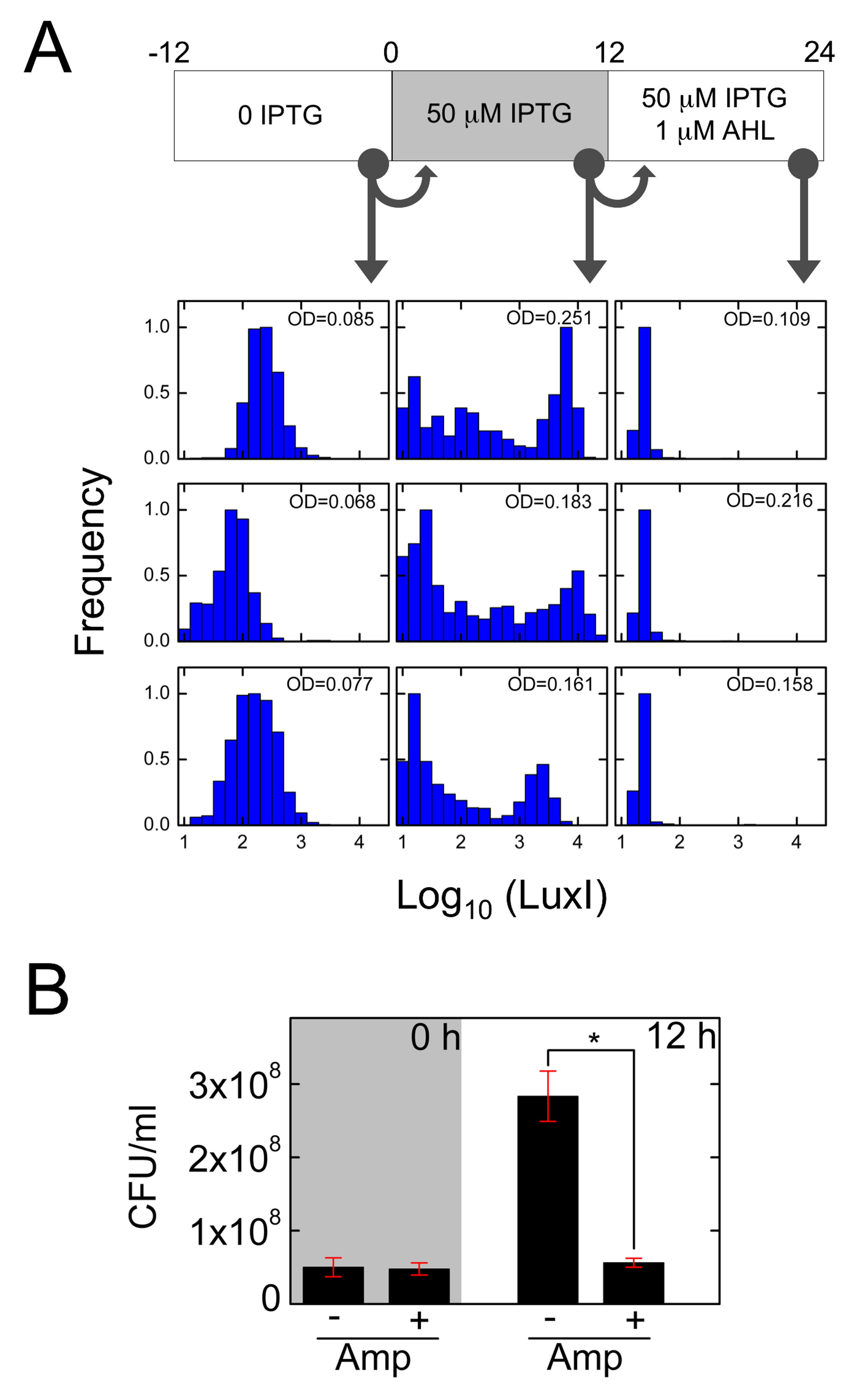

Supplement: Figure S8 — Fluorescence loss measurements. (A) We sampled Aut-IFB cells from various timepoints of the density-dependent protocol (see Materials and Methods: Autonomous loop density-dependent measurements). Cells were extracted for imaging, then re-diluted, just prior to the 0 h, 12 h, and 24 h timepoints; the OD600 values indicated correspond to pre-dilution densities. Three replicates of the same experiment are shown. Maximal LuxI::CFP fluorescence values increase throughout the first 12 h growth phase; however, a sub-population of cells show loss of fluorescence. Addition of AHL and subsequent growth to the 24 h timepoint does not lead to fluorescence recovery, indicating that the loss is irreversible. (B) Our constructs are carried on an ampicillin-resistant plasmid backbone. We measured the number of colony-forming units (CFUs) per ml of sample from the 0 h and 12 h extracts, in the presence and absence of ampicillin; errorbars represent standard error of the mean over triplicates. At 0 h all cells are ampicillin resistant (no significant difference between the two counts, p = 0.89), while at 12 h the fraction of resistant cells has fallen to less than a fifth (p = 0.003), suggesting plasmid loss is responsible for loss of fluorescence. (TIF) [file pcbi.1002361.s008.tif]
