# Supplementary material for: Prediction by Promoter Logic in Bacterial Quorum Sensing
Source: PLoS Comput Biol. 2012 Jan 19;8(1):e1002361. doi: 10.1371/journal.pcbi.1002361 (PMC3261908; doi:10.1371/journal.pcbi.1002361)
Supplement: Table S1 — List of BioBrick parts. (PDF) [file pcbi.1002361.s009.pdf]

Table S1: List of BioBrick parts.

Part	Description
BBa_R0011	pLac promoter
BBa_R0040	pTet promoter
BBa_R0062	pR promoter
BBa_B0034	Ribosome binding site
BBa_B0015	Transcription terminator
BBa_C0161	LuxI
BBa_C0062	LuxR
BBa_E0020	CFP
BBa_E0030	YFP

See the Registry of Standard Biological Parts (partsregistry.org) for details.
