# Supplementary material for: Prediction by Promoter Logic in Bacterial Quorum Sensing
Source: PLoS Comput Biol. 2012 Jan 19;8(1):e1002361. doi: 10.1371/journal.pcbi.1002361 (PMC3261908; doi:10.1371/journal.pcbi.1002361)
Supplement: Table S2 — Construct maps. (PDF) [file pcbi.1002361.s010.pdf]

Table S2: Construct maps.

ID	Description<br>*BioBrick Map*
**Sen**	pTet [LuxI::CFP]<br>*R0040.B0034.C0161.B0034.E0020.B0015*
**Rec-FF**	pLac [LuxR::YFP] pR [CFP]<br>*R0011.B0034.C0062.B0034.E0030.B0015.R0062.B0034.E0020.B0015*
**Rec-RFB**	pR [LuxR::YFP]<br>*R0062.B0034.C0062.B0034.E0030.B0015*
**Aut-RFB**	pLac [LuxI::CFP] pR [LuxR::YFP]<br>*R0011.B0034.C0161.B0034.E0020.B0015.R0062.B0034.C0062.B0034.E0030.B0015*
**Aut-IFB**	pLac [LuxR::YFP] pR [LuxI::CFP]<br>*R0011.B0034.C0062.B0034.E0030.B0015.R0062.B0034.C0161.B0034.E0020.B0015*
**Lac-CFP**	pLac [CFP]<br>*R0011.B0034.E0020.B0015*
**Lac-LuxR**	pLac [LuxR::YFP]<br>*R0011.B0034.C0062.B0034.E0030.B0015*
**Lac-LuxI**	pLac [LuxI::CFP]<br>*R0011.B0034.C0161.B0034.E0020.B0015*

Key:
Component descriptions are listed in Table S1.
[…] indicate transcription start and stop sites; double colons :: indicate polycistronic transcripts.
