# Supplementary material for: Prediction by Promoter Logic in Bacterial Quorum Sensing
Source: PLoS Comput Biol. 2012 Jan 19;8(1):e1002361. doi: 10.1371/journal.pcbi.1002361 (PMC3261908; doi:10.1371/journal.pcbi.1002361)
Supplement: Table S3 — pR promoter logic parameter values. (PDF) [file pcbi.1002361.s011.pdf]

Table S3: pR promoter logic parameter values.

Parameter	Value	StdDev	Dimension	Source
B_R	134.5	–	FL	LoE
B_I	130.2	–	FL	LoE
B_Z	0	–	FL	fixed
$\theta_R \equiv Q_Z / Q_R$	6.9	–	–	LoE
$\theta_I \equiv Q_Z / Q_I$	3.01	–	–	LoE
Q_Z	2.52E4	1.4E4	FL	PLF
m	2	–	–	fixed
n	1.45	0.22	–	PLF
β	0.0282	9.8E-3	–	PLF
$\tilde{\delta}$	4.53E-4	2.1E-4	FL^{-1}	PLF
$\tilde{\mu}$ [Sen]	2.76E-4	6.2E-5	OD^{-1} $FL^{-(1+m)/m}$	PLF
$\rho_{\max}$ [Sen]	0.1	–	OD	fixed
$\tilde{\mu}$ [Aut]	1.21E-3	–	OD^{-1} $FL^{-(1+m)/m}$	AUT
$\rho_{\max}$ [Aut]	0.05		OD	fixed

Key:

FL: Average per-pixel fluorescence intensity (Fig. 3, images)
OD: Optical density at 600 nm
LoE: Line of equivalence measurements (Fig. S2)
IND: Inducible promoter measurements (Fig. 3A,B)
PLF: Promoter logic function (Figs. 3C and S3)
AUT: Aut-RFB and Aut-IFB measurements (Fig. 5)
