# Supplementary material for: Prediction by Promoter Logic in Bacterial Quorum Sensing
Source: PLoS Comput Biol. 2012 Jan 19;8(1):e1002361. doi: 10.1371/journal.pcbi.1002361 (PMC3261908; doi:10.1371/journal.pcbi.1002361)
Supplement: Table S4 — Inducible promoter parameter values. (PDF) [file pcbi.1002361.s012.pdf]

Table S4: Inducible promoter parameter values.

Promoter	a	b	c	K
pTet	0.354	0.027	2.9	13.5 ng/ml aTc
pLac	0.063	0.051	2	104.4 μM IPTG
