# Supplementary material for: Prediction by Promoter Logic in Bacterial Quorum Sensing
Source: PLoS Comput Biol. 2012 Jan 19;8(1):e1002361. doi: 10.1371/journal.pcbi.1002361 (PMC3261908; doi:10.1371/journal.pcbi.1002361)
Supplement: Text S1 — Supporting theory, tables, and figures. (PDF) [file pcbi.1002361.s013.pdf]

Supporting Information for:

Prediction by Promoter Logic in Bacterial Quorum Sensing

Navneet Rai, Rajat Anand, Krishna Ramkumar, Varun Sreenivasan, Sugat Dabholkar, K. V. Venkatesh, and Mukund Thattai

SUPPORTING THEORY

Fluorescence backgrounds and scale factors.
Density dependence of AHL.
Modeling the promoter logic function.
Parameter estimation.
AHL and LuxR biochemistry.
Bifurcation analysis of feedback loops.
The dual positive-feedback system.
The dual positive/negative-feedback system.

SUPPORTING TABLES

Table S1: List of BioBrick parts.
Table S2: Construct maps.
Table S3: pR promoter logic parameter values.
Table S4: Inducible promoter parameter values.

SUPPORTING FIGURES

Figure S1: LuxI/LuxR quorum-sensing systems.
Figure S2: Measuring lines of equivalence.
Figure S3: Promoter logic parameter estimation.
Figure S4: AHL calibration.
Figure S5: Dynamic predictions and responses.
Figure S6: Mapping the boundary between monostable and bistable regions.
Figure S7: The dual positive-feedback system
Figure S8: Fluorescence loss measurements

SUPPORTING THEORY

Fluorescence backgrounds and scale factors. We want to relate the units of three different quantities: the passive reporter CFP (Y_Z); LuxR::YFP (Y_R); and LuxI::CFP (Y_I). To do this, we express each of them in turn (Lac-CFP, Lac-LuxR, Lac-LuxI; see Table S2) downstream of the pLac promoter and compare their levels at identical IPTG concentrations. Let the transcription rate be represented as $\alpha_\bullet$ (defined with respect to the maximal level of the function f in Eq. 2):

$$\frac{dY_\bullet}{dt} = \gamma_\bullet\left[Q_\bullet \alpha_\bullet - Y_\bullet\right] \quad \Rightarrow \quad Y_\bullet = Q_\bullet \alpha_\bullet, \qquad \text{Eq. S1}$$

where, as in the main text, the parameters $Q_\bullet$ are protein production rates per transcript, scaled by the protein decay rates $\gamma_\bullet$. If we compare the output of all three proteins at identical IPTG concentrations, they will all have the same value of $\alpha_\bullet$. This implies

$$Y_Z = \theta_R\, Y_R = \theta_I\, Y_I, \qquad \text{where} \quad \theta_R \equiv Q_Z / Q_R, \quad \theta_I \equiv Q_Z / Q_I. \qquad \text{Eq. S2}$$

In practice our actual measured quantities $Y_\bullet^\#$ will also include background fluorescence levels, represented $Y_\bullet^\# = Y_\bullet + B_\bullet$. This gives us the following equations for *lines of equivalence*:

$$Y_Z^\# = \theta_R Y_R^\# + (B_Z - \theta_R B_R) = \theta_I Y_I^\# + (B_Z - \theta_I B_I). \qquad \text{Eq. S3}$$

These slope and intercept values can be determined by an affine fit (Fig. S2). The slopes correspond precisely to the term required on the right-hand side of Eq. 5. The background values $B_\bullet$ cannot be specified uniquely. However, as both y-intercepts are seen to be negative, the lowest possible background values can be found by setting $B_Z = 0$ and calculating the resulting B_R and B_I. In the main text, data include background values unless otherwise indicated; in the discussion that follows, symbols without hashes indicate quantities with backgrounds subtracted. Table S3 gives fitted values (key: LoE).

We will sometimes want to express transcription rates at pTet and pLac as functions of inducer concentrations (either aTc or IPTG). We can fit their induction profiles (e.g. Fig. 3A,B), with backgrounds subtracted, to Hill functions:

$$Y_{\bullet}^{\#} - B_{\bullet} = Y_{\bullet} = Q_{\bullet}\alpha_{\bullet}, \qquad \text{with} \qquad \alpha_{\bullet} = a\left(b + (1-b)\frac{x^c}{K^c + x^c}\right). \qquad \text{Eq. S4}$$

Here, the quantity x represents either [IPTG] in μM or [aTc] in ng/ml or, for pLac and pTet respectively. We determined parameter values by least-squares fitting for the induction constructs pTet [LuxI::CFP] and pLac [LuxR::YFP] (see Table S2). All parameters were varied for the pTet construct; the Hill coefficient c was set to 2 for the pLac construct. Table S4 gives fitted values.

Density dependence of AHL. Suppose cells are growing in a niche of volume V; AHL is synthesized by intracellular LuxI at specific rate k but diffuses rapidly across the cell membrane; and AHL is removed from this niche, or equivalently it is degraded, with rate constant γ_ϕ. In the limit that AHL diffusion is much more rapid than degradation, we can assume that its intracellular and extracellular concentrations are equal; experiments using radio-labeled AHL have shown that the ratio of intracellular to extracellular concentrations typically lies in the range 0.9 – 1.1 [37]. The accumulation of AHL is then described by the following equation:

$$\frac{d\phi}{dt} = k\frac{V_c}{V}\rho Y_I - \gamma_\phi \phi, \qquad \text{Eq. S5}$$

where ϕ is the AHL concentration, Y_I is the intracellular LuxI concentration, ρ is the cell number density, and V_c is the volume of a single cell. The ratio V_c/V accounts for the fact that AHL is synthesized only in the intracellular volume, but diffuses throughout the niche [37]. Now consider the thought experiment in which cell growth is clamped at some nominal density ρ. AHL levels would reach a steady state, found by setting $d\phi/dt = 0$:

$$\phi = \frac{k}{\gamma_\phi}\frac{V_c}{V}\rho Y_I \equiv \mu\rho Y_I, \qquad \text{Eq. S6}$$

as shown in Eq. 1 of the main text. Next consider the case in which cell density is increasing exponentially, so $\rho(t) = \rho_0 e^{\gamma_c t}$. If LuxI levels are given by $Y_I(t)$, we can integrate Eq. S5 to obtain:

$$\phi(t) = \phi(0)e^{-\gamma_\phi t} + \frac{kV_c\rho_0}{V(\gamma_c + \gamma_\phi)}\left([Y_I(t)e^{\gamma_c t} - Y_I(0)e^{-\gamma_\phi t}] - e^{-\gamma_\phi t}\int_o^t Y_I'(\tau)e^{(\gamma_c+\gamma_\phi)\tau}d\tau\right). \qquad \text{Eq. S7}$$

where $Y_I'(\tau)$ represents a time-derivative evaluated at time τ. In the sender-cell experiment where LuxI levels are held fixed and $\phi(0)=0$, the solution is:

$$\phi(t)=\frac{kV_c}{V(\gamma_c+\gamma_\phi)}\rho_0 e^{\gamma_c t}Y_I \equiv \mu'\rho Y_I. \qquad \text{Eq. S8}$$

That is, the AHL concentration is proportional to the cell density at the time of measurement, as in Eq. 1 of the main text (but with a different constant of proportionality than in the growth-clamped case of Eq. S6). Thus the same AHL-density proportionality law arises in the static growth-clamped case and in the dynamic exponential growth case.

Modeling the promoter logic function. To study the range of possible steady-states of Eq. 4 under growth-clamped conditions, we must first specify the form of the function $f(\phi, Y_R)$. We start with the proportionality condition Eq. 1, which relates the AHL concentration (ϕ) to the current cell density (ρ) and intracellular LuxI levels (Y_I):

$$\phi=\mu\rho Y_I. \qquad \text{Eq. S9}$$

AHL binds to and activates LuxR, which we can describe by a Hill equation with basal activity:

$$\frac{Y_R^*}{Y_R}=\delta+(1-\delta)\frac{\phi^m}{K_\phi^m+\phi^m}\equiv g(\phi), \qquad \text{Eq. S10}$$

where Y_R is the total concentration of LuxR, and Y_R^* is the concentration of its active form. In turn, AHL-bound LuxR activates transcription at promoter pR; we can describe this process, too, with a Hill equation:

$$f(\phi,Y_R)=\beta+(1-\beta)\frac{Y_{R^*}^n}{K_R^n+Y_{R^*}^n}=\beta+(1-\beta)\frac{Y_R^n\, g(\mu\rho Y_I)^n}{K_R^n+Y_R^n\, g(\mu\rho Y_I)^n}, \qquad \text{Eq. S11}$$

where the maximum value of f is explicitly set to one, because it defines the unit rate of transcription (Eq. 2). The parameterization of f in terms of nested Hill equations, although being a convenient way to capture the cooperativity of AHL-LuxR binding and LuxR-DNA binding [12] is only one among several reasonable options. In the main text we determine this function by direct experimental measurement, rather than relying on any specific parameterization.

Parameter estimation. A biochemically-motivated parameterization is not strictly necessary, but it is useful to the extent that meaningful parameter values can be estimated from measured data. Applying Eq. 4, the CFP output from the feedforward measurement that is used to determine the PLF is:

$$Y_Z = Q_Z f(\mu\rho\overline{Y}_I, \overline{Y}_R), \qquad \text{Eq. S12}$$

where symbols with overbars indicate fixed input values. Assume for the moment that $\phi << K_\phi$ (we will later show this is the case; see Eq. S15). Using Eq. S10, we can then approximate Y_R^* as

$$\frac{Y_R^*}{K_R} = \overline{Y}_R \frac{g(\mu\rho\overline{Y}_I)}{K_R} \approx \overline{Y}_R(\delta/K_R + (1-\delta)(\mu/K_\phi)^m/K_R \cdot \rho^m\overline{Y}_I^m). \qquad \text{Eq. S13}$$

Setting $\tilde{\delta} \equiv \delta/K_R$ and $\tilde{\mu}^m \equiv (1-\delta)(\mu/K_\phi)^m/K_R$, this gives us the final functional form:

$$\frac{Y_Z}{Q_Z} = \beta + (1-\beta)\frac{\overline{Y}_R^n(\tilde{\delta} + (\tilde{\mu}\rho\overline{Y}_I)^m)^n}{1 + \overline{Y}_R^n(\tilde{\delta} + (\tilde{\mu}\rho\overline{Y}_I)^m)^n} \qquad \text{Eq. S14}$$

We can now estimate parameter values. The nominal cell density for the sender-receiver experiment is $\rho = 0.1$; this leaves 6 independent parameters that must be estimated. We performed nonlinear least-squares fitting using the MATLAB *fminsearch* function (Mathworks). The dataset from the PLF has 42 measured values of Y_Z, for all combinations of the 7 measured values of Y_R and the 6 measured values of Y_I. We fit these data to the form of Eq. S14, doing a bounded parameter search from random initial conditions, with chi-square calculated in log space assuming uniform measurement errors. The Hill coefficient m invariably increased without bound; to ensure numerical convergence, we clamped it at $m = 2$ (Fig. S3A). This procedure robustly converged to a set of best-fit parameter values (Fig. S3B). We then ran Monte Carlo trials, generating synthetic datasets from Eq. S14 with the best-fit parameters, introducing Gaussian multiplicative noise for the 42 data points from standard errors of measurement, and re-running the least-squares routine [42]. This allowed us to estimate confidence intervals on the parameter values, as the standard deviation of best-fit values over 1000 Monte Carlo trials. This fit has a chi-square probability Q = 0.80 (Fig. S3C). Table S3 gives fitted values (key: PLF, with $\tilde{\mu}$ explicitly listed as $\tilde{\mu}$ [Sen] to distinguish it from the value $\tilde{\mu}$ [Aut] which is determined from the autonomous loop measurements).

AHL and LuxR biochemistry. Urbanowski *et al.* [12] have used biochemical analyses to characterize AHL-LuxR binding, and the binding of activated LuxR to pR. We can compare the results of our fitting procedure to their direct measurements.

AHL-LuxR binding. In our parameterized model, we set the value $m = 2$ by hand. Our AHL titration curve has a broad range of uncertainty in its best-fit Hill coefficient: $m = 1.9 \pm 0.5$ (Fig. S4C). For comparison, we show the gel-shift measurement of Urbanowski *et al.* with the same Hill coefficient (Fig. S4D). The results are reasonable, though not the best fit; Urbanowski *et al.* obtain a graphical estimate of $m = 1$. We find a half-saturation AHL concentration of ~ 825 nM, while Urbanowski *et al.* report a value of ~ 85 nM. This discrepancy is likely due to AHL degradation over the 12 h duration of our experiment (see Materials and Methods: AHL Calibration).

LuxR-DNA binding. For LuxR-DNA binding, we estimate a Hill coefficient of $n = 1.45$; for comparison we show data from the protection assay of Urbanowski *et al.*, which is in excellent agreement with the same Hill coefficient (Fig. S4E). Urbanowski *et al.* report that the half-saturation concentration for LuxR-DNA binding is ~ 0.1 nM. When we consider that 1 nM corresponds to approximately 1 molecule in a μm-sized bacterial cell, and that the constructs used in our experiments are expressed from high-copy plasmids, we can safely assume that $Y_R >> K_R$. In turn, this means that only a small fraction of LuxR is active by the time LuxR-DNA binding is saturated; further increases in AHL levels cannot affect system behavior, and can effectively be ignored. This justifies the approximation made in Eq. S13:

$$Y_R >> K_R \quad \Rightarrow \quad g(\phi) << 1 \quad \Leftrightarrow \quad \phi << K_\phi. \qquad \text{Eq. S15}$$

Bifurcation analysis of feedback loops. In the feedback systems of Eq. 4, the protein in feedback ($Y_\bullet$) is expressed from pR with transcription rate f, while the fixed input protein ($\overline{Y}_\bullet$) is expressed from some constitutive promoter pX. Suppose the pX has a transcription rate α (where for convenience we have dropped the subscript that was explicit in Eq. S1). It is convenient to introduce the following dimensionless measures of protein levels:

$$y_R \equiv Y_R / Q_R, \qquad y_I \equiv Y_I / Q_I. \qquad \text{Eq. S16}$$

We can then express the steady-state conditions of the constant-density thought experiment as algebraic equations in either y_R or y_I.

LuxR feedback: Eq. S17

$$\frac{1}{\gamma_I}\frac{dY_I}{dt} = Q_I\alpha - Y_I \quad \overset{SS}{\Rightarrow} \quad \overline{Y}_I = Q_I\alpha$$

$$\frac{1}{\gamma_R}\frac{dY_R}{dt} = Q_R f(\mu\rho\overline{Y}_I, Y_R) - Y_R \quad \overset{SS}{\Rightarrow} \quad y_R = \beta + (1-\beta)\frac{(Q_R \cdot y_R)^n(\tilde{\delta} + (\tilde{\mu}\rho Q_I \cdot \alpha)^m)^n}{1 + (Q_R \cdot y_R)^n(\tilde{\delta} + (\tilde{\mu}\rho Q_I \cdot \alpha)^m)^n}$$

LuxI feedback: Eq. S18

$$\frac{1}{\gamma_R}\frac{dY_R}{dt} = Q_R\alpha - Y_R \quad \overset{SS}{\Rightarrow} \quad \overline{Y}_R = Q_R\alpha$$

$$\frac{1}{\gamma_I}\frac{dY_I}{dt} = Q_I f(\mu\rho Y_I, \overline{Y}_R) - Y_I \quad \overset{SS}{\Rightarrow} \quad y_I = \beta + (1-\beta)\frac{(Q_R \cdot \alpha)^n(\tilde{\delta} + (\tilde{\mu}\rho Q_I \cdot y_I)^m)^n}{1 + (Q_R \cdot \alpha)^n(\tilde{\delta} + (\tilde{\mu}\rho Q_I \cdot y_I)^m)^n}$$

The condition of Eq. S15 can be expressed as $Q_R\beta >> K_R$ for LuxR feedback, or $Q_R\alpha >> K_R$ for LuxI feedback. As long as these are satisfied, Eqs. S17 and S18 are valid throughout their domains. Holding all parameter values except for ρ fixed, Eqs. S17 and S18 can be used to solve for either y_R or y_I as a function of cell density, up to some terminal value $\rho_{\max}$. This is precisely the DDR of the LuxR-feedback or LuxI-feedback system, examples of which are shown in Fig. 1A. Now, for each combination of parameters $\{\alpha, n, m, \beta, \tilde{\delta}, \rho_{\max}, \tilde{\mu}Q_I, Q_R\}$ we can classify the resulting DDR into one of 4 categories (monostable type M; bistable types B+, B±, B-) and can numerically identify the parametric boundaries that separate these functionally distinct response types. Figure 6 shows a 2-dimensional slice of this bifurcation diagram for LuxR-feedback and LuxI-feedback systems in $\{\alpha, n\}$ space, with the remaining parameters set to the values shown in Table S3 (with $\tilde{\mu}$ and $\rho_{\max}$ from autonomous loop experiments). In fact, the DDR bifurcation diagrams for LuxR-feedback and LuxI-feedback systems are *always* qualitatively identical to those shown in Fig. 6, though with different numerical values for transitions as parameters are varied. We now sketch the proof of this.

The key feature of the bifurcation diagram in $\{\alpha, n\}$ space is the line of critical points that separates the type M and type B regions. Transitions to the right of this line (between types B+, B±, and B-) arise from the fact that cell density cannot exceed the value $\rho_{\max}$: transitions from B+ to B± to B- occur at successively smaller values of α as $\rho_{\max}$ is increased, and in the limit $\rho_{\max} \to \infty$ only the type B+ region remains (Fig. S6). The point where the B+, B±, and B- regions meet satisfies a criticality condition;

we can trace out the critical line C by solving this condition as $\rho_{\max}$ is varied from 0 to ∞. Let time-evolution equations Eq. S17 and S18 be represented as

$$\frac{dy_\bullet}{dt} = g(y_\bullet; \rho, \alpha, n, ...), \qquad \text{Eq. S19}$$

where we have explicitly highlighted parameters of special interest. The criticality conditions are:

$$g(y_\bullet; \rho, \alpha, n, ...) = \frac{\partial}{\partial y_\bullet} g(y_\bullet; \rho, \alpha, n, ...) = \frac{\partial^2}{\partial y_\bullet^2} g(y_\bullet; \rho, \alpha, n, ...) = 0. \qquad \text{Eq. S20}$$

These can be used to solve for $y_\bullet$ and two other unknowns: $\alpha^*(\rho,...), n^*(\rho,...), y_\bullet^*(\rho,...)$, where the asterisk designates critical values. The first two terms trace the line of critical points in $\{\alpha, n\}$ space parameterized by ρ, keeping all other parameters fixed.

Consider the LuxR-feedback system, Eq. S17:

$$g(y_R) = \beta + (1-\beta)\frac{x_R^n \cdot y_R^{\ n}}{1 + x_R^n \cdot y_R^n} - y_R, \text{ with } x_R \equiv Q_R(\tilde{\delta} + (\tilde{\mu}\rho Q_I \alpha)^m). \qquad \text{Eq. S21}$$

Applying conditions S19, we find:

$$\begin{aligned} x_R^* &= Q_R(\tilde{\delta} + (\tilde{\mu}\rho Q_I \alpha^*)^m) = \beta^{-\sqrt{\beta}/(1+\sqrt{\beta})} \\ n^* &= (1+\sqrt{\beta})/(1-\sqrt{\beta}) \\ y_R^* &= \sqrt{\beta} \end{aligned} \qquad \text{Eq. S22}$$

This is a degenerate case in which n^* is independent of ρ, and increases monotonically from 1 to ∞ as β is varied from 0 to 1. x_R^* is a peaked function of β bounded between 1 and 1.75. If $Q_R\tilde{\delta} < x_R^*$, we can always find α^* in terms of ρ, giving us a vertical line of critical points; this happens to be the case for our fitted parameters. As $Q_R\tilde{\delta}$ grows larger, the threshold value of ρ required to activate the system in the bistable region drops until, at $Q_R\tilde{\delta} = x_R^*$, the system is either monostable for $n < n^*$, or constitutively active for $n > n^*$. For our fitted parameter values, we calculate $n^* = 1.40$, as seen in Fig. 6A.

We next examine the LuxI-feedback system, Eq. S18:

$$g(y_I) = \beta + (1-\beta)\frac{(Q_R\alpha)^n(\tilde{\delta} + (\tilde{\mu}\rho Q_I y_I)^m)^n}{1 + (Q_R\alpha)^n(\tilde{\delta} + (\tilde{\mu}\rho Q_I y_I)^m)^n} - y_I. \qquad \text{Eq. S23}$$

In general, the line of critical points will be some curve $\{\alpha^*(\rho,...), n^*(\rho,...)\}$ whose position depends on other parameter values, and must be found numerically. However, it is illuminating to ask what happens in the limit $\alpha \to 0$. Naively this seems to imply basal transcription; however, no matter how small α becomes there will be some value of ρ at which the system can be induced (assuming throughout that condition Eq. S15 is valid). Therefore, we must take the joint limit

$$\underset{\alpha\to 0,\rho\to\infty}{\text{Lim}} \alpha\rho^m \equiv \omega^m. \qquad \text{Eq. S24}$$

Then we can write

$$\underset{\alpha\to 0,\rho\to\infty}{\text{Lim}} g(y_I) = \beta + (1-\beta)\frac{x_I^{mn}\cdot y_I^{mn}}{1 + x_I^{mn}\cdot y_I^{mn}} - y_I, \text{ with } x_I \equiv Q_R^{1/m} Q_I \tilde{\mu}\omega. \qquad \text{Eq. S25}$$

This is solved similarly to the LuxR-feedback case, and we find:

$$\begin{aligned} x_I^* &= Q_R^{1/m} Q_I \tilde{\mu}\omega = \beta^{-\sqrt{\beta}/(1+\sqrt{\beta})} \\ mn^* &= (1+\sqrt{\beta})/(1-\sqrt{\beta}) \\ y_I^* &= \sqrt{\beta} \end{aligned} \qquad \text{Eq. S26}$$

The critical value n^* for LuxI feedback can be lower than that for the LuxR-feedback case because it is only the product mn^* that matters. And unlike in the LuxR-feedback case, the equation for x_I^* can always be satisfied by some positive value of ω. For our fitted parameters, we have $n^* = 0.70$ on the $\alpha = 0$ axis, as seen in Fig. 6B.

The dual positive-feedback system. In a direct dual feedback system, both LuxI and LuxR would be expressed downstream of pR, and we would therefore have $y_R = y_I = y$ in steady-state. More generally, we could imagine that the two genes are expressed at a fixed ratio (or equivalently, have distinct

translation rates): $y_R / A = y_I = y$. Under these conditions, the steady-states are given by solutions of the following equation:

$$y = \beta + (1-\beta)\frac{(Q_R A y)^n (\tilde{\delta} + (\tilde{\mu}\rho Q_I y)^m)^n}{1 + (Q_R A y)^n (\tilde{\delta} + (\tilde{\mu}\rho Q_I y)^m)^n} \qquad \text{Eq. S27}$$

We can calculate the DDR as before, giving the output transcription rate as a function of cell density (Fig. S7).

The dual positive/negative-feedback system. We model the dual positive/negative-feedback system with the following ordinary differential equations:

$$\begin{aligned}
\frac{1}{\gamma_R}\frac{dY_R}{dt} &= Q_R f(\mu\rho Y_I, Y_R) - Y_R \\
\frac{1}{\gamma_{Lac}}\frac{dY_{Lac}}{dt} &= Q_{Lac} f(\mu\rho Y_I, Y_R) - Y_{Lac} \\
\frac{1}{\gamma_I}\frac{dY_I}{dt} &= Q_I g(Y_{Lac}) - Y_I
\end{aligned} \qquad \text{Eq. S28}$$

where Y_{Lac} is the concentration of the repressor LacI; we set the units of Y_{Lac} by defining $Q_{Lac} = Q_R$. For the function $f(\cdot)$ we use parameter values from the autonomous feedback loop experiments (Table S3), and set $\rho = 0.185$ to match the cell density in the nitrogen-limited chemostat. The function $g(Y_{Lac})$ describes the transcription rate of LuxI from the pLac promoter. In principle this function can itself be experimentally determined; here we use a previously described functional form [25]:

$$g(Y_{Lac}) = a_{Lac}\frac{K_{Lac}}{K_{Lac} + Y_{Lac}}. \qquad \text{Eq. S29}$$

The constant a_{Lac} has already been measured (Table S4), so only the half-saturation parameter K_{Lac} must be set. Finally, our polycistronic reporter experiments do not allow us to directly measure response rates; for simplicity, we assume that $\gamma_R = \gamma_{Lac} = 1$ (thus setting the unit of time) and leave γ_I as a free parameter. The dynamical equations for Y_R and Y_{Lac} are identical in this setting, so we simply assume that $Y_{Lac} = Y_R$ at all times, resulting in a two-dimensional system of ODEs. We numerically solve these equations using the MATLAB *ode45* solver (Mathworks). Sample timecourses are shown in Fig. 7 of the main text, for the value $K_{Lac} = 25$, and the two cases $\gamma_I = 0.1, 0.01$.

Table S1: List of BioBrick parts.

Part	Description
BBa_R0011	pLac promoter
BBa_R0040	pTet promoter
BBa_R0062	pR promoter
BBa_B0034	Ribosome binding site
BBa_B0015	Transcription terminator
BBa_C0161	LuxI
BBa_C0062	LuxR
BBa_E0020	CFP
BBa_E0030	YFP

See the Registry of Standard Biological Parts (partsregistry.org) for details.

Table S2: Construct maps.

**ID**	Description<br>*BioBrick Map*
**Sen**	pTet [LuxI::CFP]<br>*R0040.B0034.C0161.B0034.E0020.B0015*
**Rec-FF**	pLac [LuxR::YFP] pR [CFP]<br>*R0011.B0034.C0062.B0034.E0030.B0015.R0062.B0034.E0020.B0015*
**Rec-RFB**	pR [LuxR::YFP]<br>*R0062.B0034.C0062.B0034.E0030.B0015*
**Aut-RFB**	pLac [LuxI::CFP] pR [LuxR::YFP]<br>*R0011.B0034.C0161.B0034.E0020.B0015.R0062.B0034.C0062.B0034.E0030.B0015*
**Aut-IFB**	pLac [LuxR::YFP] pR [LuxI::CFP]<br>*R0011.B0034.C0062.B0034.E0030.B0015.R0062.B0034.C0161.B0034.E0020.B0015*
**Lac-CFP**	pLac [CFP]<br>*R0011.B0034.E0020.B0015*
**Lac-LuxR**	pLac [LuxR::YFP]<br>*R0011.B0034.C0062.B0034.E0030.B0015*
**Lac-LuxI**	pLac [LuxI::CFP]<br>*R0011.B0034.C0161.B0034.E0020.B0015*

Key:
Component descriptions are listed in Table S1.
[…] indicate transcription start and stop sites; double colons :: indicate polycistronic transcripts.

Table S3: pR promoter logic parameter values.

Parameter	Value	StdDev	Dimension	Source
B_R	134.5	–	FL	LoE
B_I	130.2	–	FL	LoE
B_Z	0	–	FL	fixed
$\theta_R \equiv Q_Z / Q_R$	6.9	–	–	LoE
$\theta_I \equiv Q_Z / Q_I$	3.01	–	–	LoE
Q_Z	2.52E4	1.4E4	FL	PLF
m	2	–	–	fixed
n	1.45	0.22	–	PLF
β	0.0282	9.8E-3	–	PLF
$\tilde{\delta}$	4.53E-4	2.1E-4	FL^{-1}	PLF
$\tilde{\mu}$ [Sen]	2.76E-4	6.2E-5	OD^{-1} $FL^{-(1+m)/m}$	PLF
ρ_{max} [Sen]	0.1	–	OD	fixed
$\tilde{\mu}$ [Aut]	1.21E-3	–	OD^{-1} $FL^{-(1+m)/m}$	AUT
ρ_{max} [Aut]	0.05		OD	fixed

Key:

FL: Average per-pixel fluorescence intensity (Fig. 3, images)
OD: Optical density at 600 nm
LoE: Line of equivalence measurements (Fig. S2)
IND: Inducible promoter measurements (Fig. 3A,B)
PLF: Promoter logic function (Figs. 3C and S3)
AUT: Aut-RFB and Aut-IFB measurements (Fig. 5)

Table S4: Inducible promoter parameter values.

Promoter	a	b	c	K
pTet	0.354	0.027	2.9	13.5 ng/ml aTc
pLac	0.063	0.051	2	104.4 μM IPTG

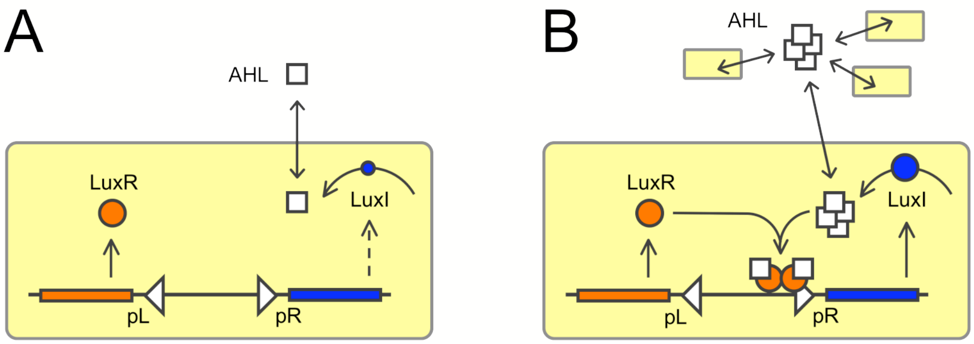

Figure S1: LuxI/LuxR quorum-sensing systems. LuxI (blue circle) is an enzyme that synthesizes acyl-homoserine lactone (AHL; white square). LuxR (orange circle) is a transcriptional activator. (A) At low cell densities, LuxR is expressed at high levels from the pL promoter, while LuxI is expressed at a basal level from the pR promoter. AHL is synthesized at low levels, and diffuses freely across the cell membrane. LuxR remains in an inactive form. (B) At high cell densities, the aggregate synthesis of AHL from many cells drives up its extracellular and intracellular concentration, promoting LuxR-AHL binding. AHL-bound LuxR activates transcription of LuxI at the pR promoter, driving a positive-feedback loop.

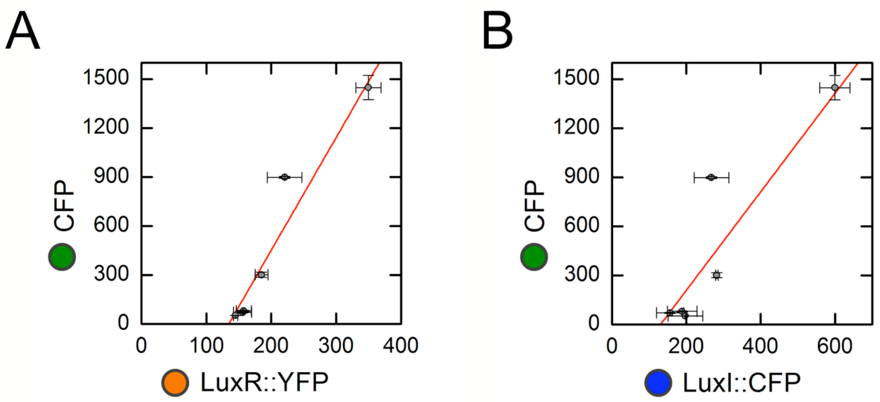

Figure S2: Measuring lines of equivalence. We determined CFP, LuxR::YFP, and LuxI::CFP values for proteins expressed from pLac with IPTG = [0 5 10 50 100 500] μM. Each datapoint gives either the (A) LuxR::YFP or (B) LuxI::CFP level against the corresponding CFP level at equal IPTG concentrations; error bars represent standard errors of measurement over replicates. The lines of equivalence (red) are determined by affine fits.

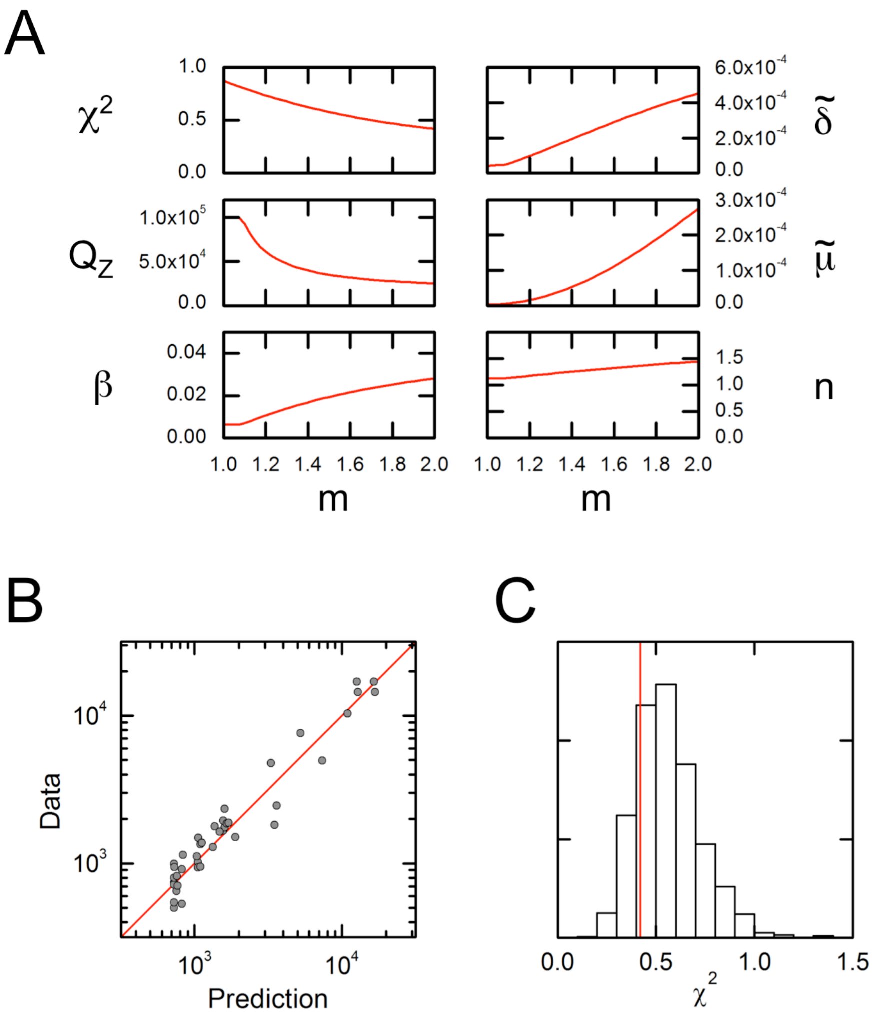

Figure S3: Promoter logic parameter estimation. (A) We estimated the parameters of Eq. 6 / Eq. S14 by non-linear least-squares fitting. We observed for an unconstrained fit that the value of the LuxR-AHL binding Hill coefficient m increased without bound; but if the value of m was fixed, the algorithm robustly converged to a set of best-fit parameters. Here we show fitted parameter values as a function of m. The chi-square error (top left graph) decreases monotonically with m; this underlies the numerical instability. Throughout the paper, parameter values are those determined for m = 2. The value of the LuxR-DNA binding Hill coefficient n is only weakly dependent on m (bottom right graph). (B) Predicted vs. observed CFP values for the 42 datapoints of the PLF, from a 5-parameter fit. (C) The histogram shows the distribution of chi-square values found for 1000 Monte Carlo trials using synthetic datasets. A fraction Q = 0.8 of these values are greater than value from the actual fit (vertical red line), showing that the deviations in Fig. S3B are within measurement error.

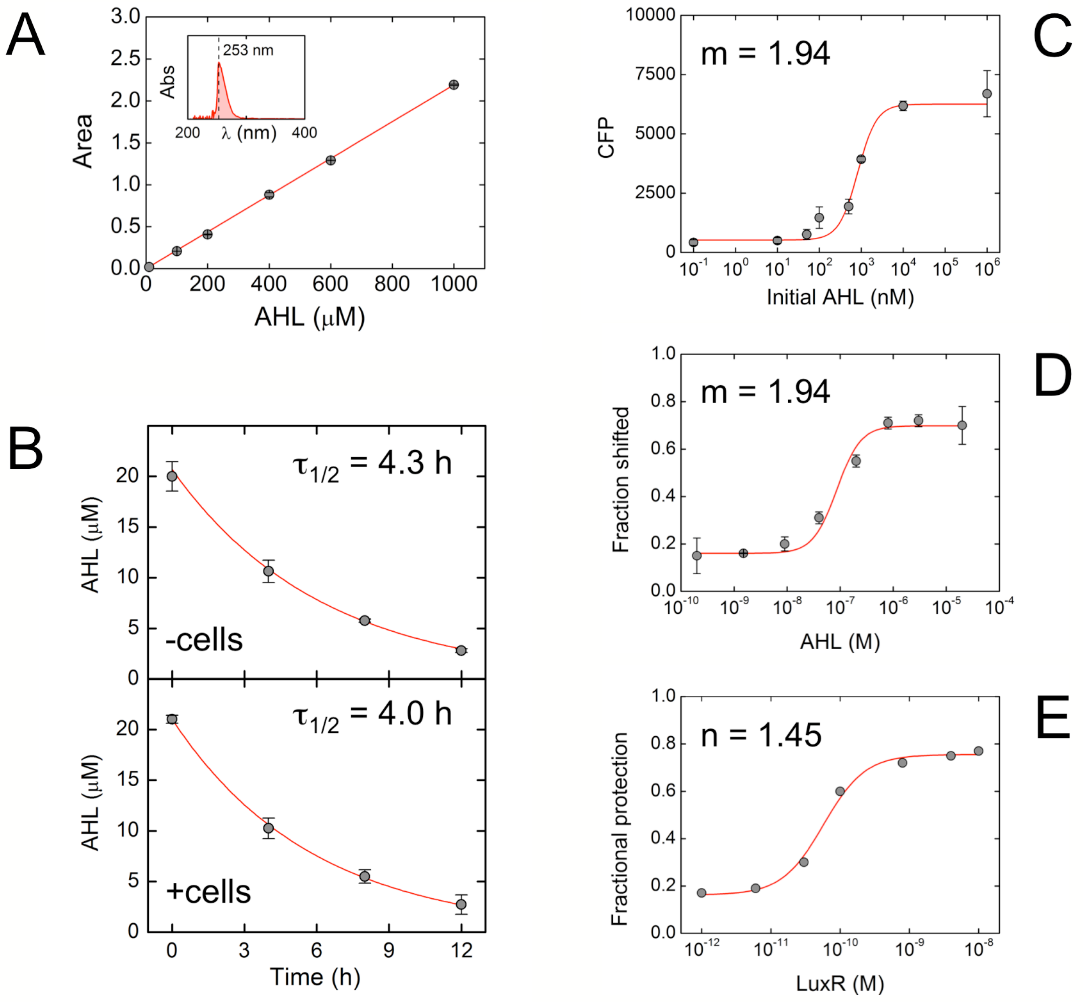

Figure S4: AHL calibration. (A) The area under the curve from HPLC measurements of absorption at λ = 253 nm, plotted against synthetic AHL concentration. The inset shows the absorption peak. (B) AHL decay measured using HPLC. The exponential fit shows that AHL decays with a half-life of ~ 4 h, independent of the presence or absence of cells in the medium. (C) Titration: the CFP levels of Rec-FF cells (with LuxR induced using 500 μM IPTG) plotted against the initial levels of synthetic AHL in the medium. The curve shows a Hill fit, with the best fit Hill coefficient m = 1.94 ± 0.5. (D) Data from gel-shift experiments of LuxR-to-AHL binding for 3.5 nm total LuxR, as a function of AHL levels. The curve shows a fit with the Hill coefficient fixed at m = 1.94. Datapoints estimated graphically from figures in Urbanowski *et al.* [12]. (E) Data from DNA protection experiments probing the binding of LuxR-AHL to DNA as a function of LuxR levels, when AHL is in excess (10 μM). The curve shows a fit with the Hill coefficient fixed at n = 1.45, as estimated from our PLF measurements. Datapoints estimated graphically from figures in Urbanowski *et al.* [12].

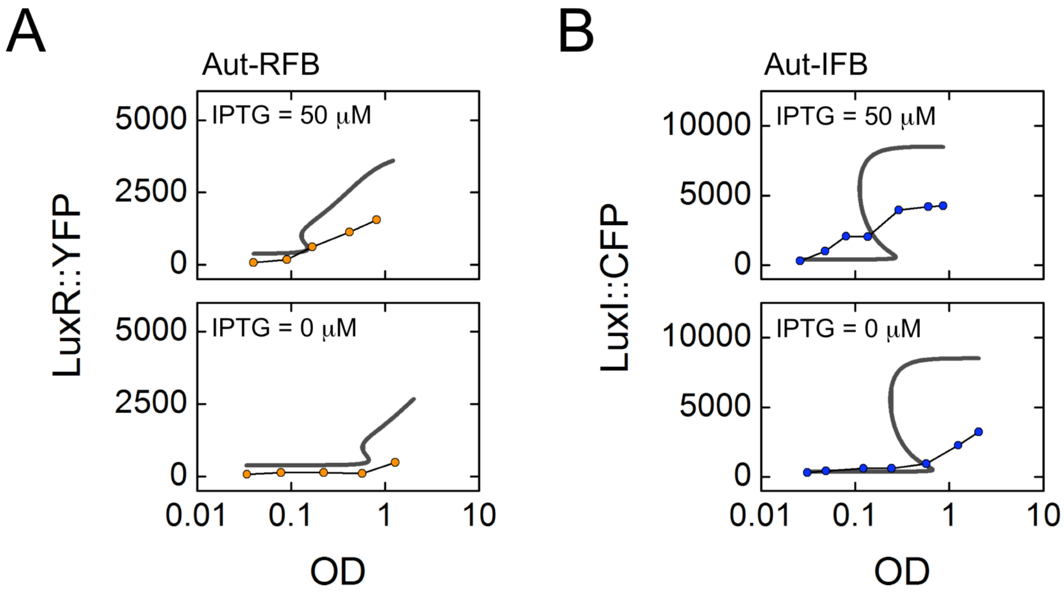

Figure S5: Dynamic predictions and responses. We predicted the entire density-dependent response of the two autonomous loop constructs (using Eqs. S17 and S18, with parameters from Table S3), starting from low density and going up to the carrying capacity of our media (OD_{600} ~ 1). As expected given the high rates of change of cell density under these conditions, the observed feedback response lags the predicted DDR at all times. Nevertheless, the predictions correctly capture how changes in the regulator level accelerate the induction dynamics. Grey curves show the DDR predicted from Eqs. S17 and S18, with parameters from Table S4. In principle the parameter $\tilde{\mu}$ should be re-calculated for these new high-density growth conditions, but we have used $\tilde{\mu}$ [Sen] directly (Table S3). Datapoints show the observed responses for (A) the Aut-RFB system and (B) the Aut-IFB system. We determined responses at two different IPTG concentrations (hence two different levels of the regulator LuxI::CFP or LuxR::YFP, respectively). Measurements were made at 2 h intervals until the cultures entered stationary phase.

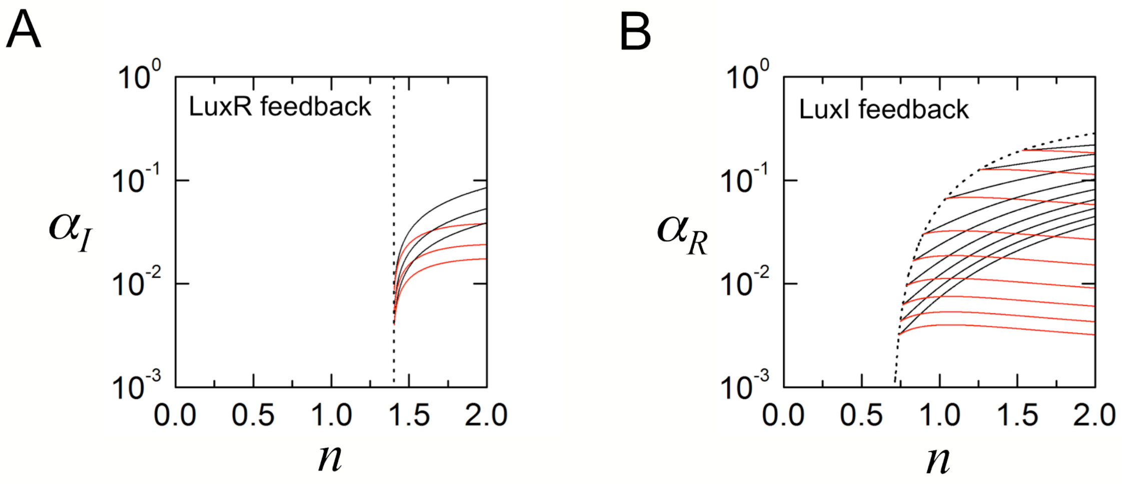

Figure S6: Mapping the boundary between monostable and bistable regions. Using autonomous loop parameters from Table S3 and a fixed value of cell density ρ, we can find the regions of $\{\alpha, n\}$ space that admit bistable solutions (regions within the taper emanating from a critical point, bounded by a set of black and red curves). As ρ is increased up to the level $\rho_{\max}$, these tapers move toward lower values of α. Any given point will transition from the un-induced (below taper) to the bistable (within taper) to the fully induced (above taper) regions, thus mapping out the DDR as a function of ρ. Once we reach $\rho_{\max}$, any point above the taper would have already been induced (B+); any point still inside the taper would be hysteretic (B±); and any point below the taper would be un-induced (B-); Fig. 6 was generated for $\rho_{\max}$ = 0.05. By tracing out the critical points as cell density is increased from 0 to ∞, we can find the line that separates the type M and type B regions.

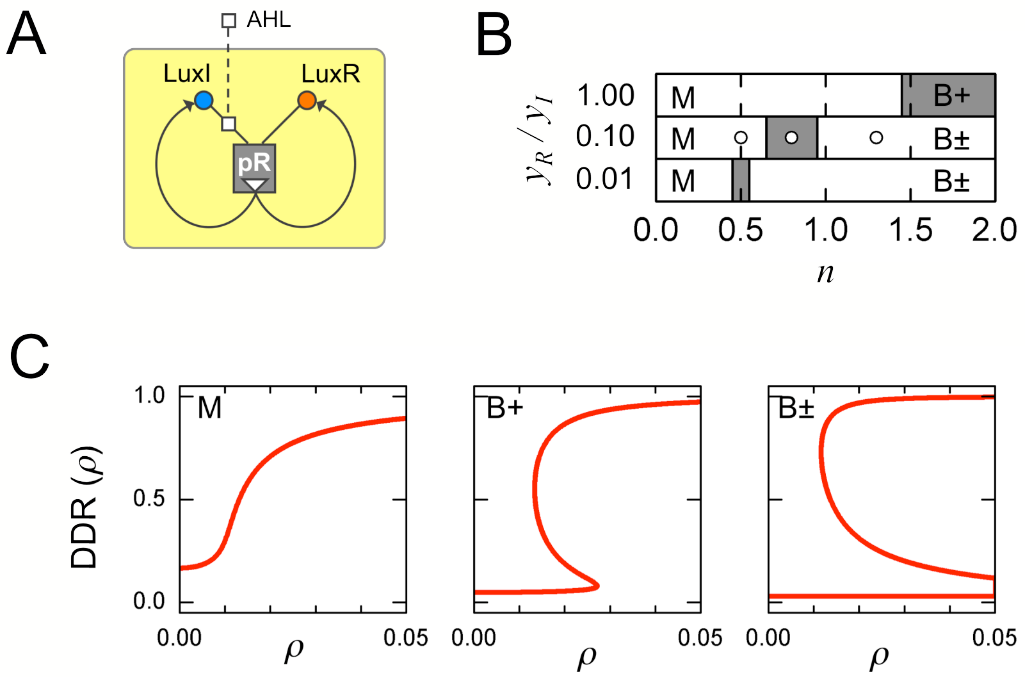

Figure S7: The dual positive-feedback system. (A) Dual positive feedback is achieved by placing both LuxR and LuxI downstream of pR. This system has no regulator, but is still sensitive to extracellular AHL levels. (B) We model the system using the autonomous loop parameters from Table S3. We further allow the relative translational efficiencies of LuxI and LuxR to be tuned: the condition $y_R / y_I = 1$ means we use the directly measured translation rates, while $y_R / y_I = 0.1$ is equivalent to LuxR having a 10-times reduced translation rate (Eq. S27). As in Fig. 1 of the main text, we solve for the density-dependent response (DDR) of the system for various values y_R / y_I, and of the Hill coefficient n. As n is increased, the system moves from type M (white), through type B+ (grey) and eventually to type B± (white). (C) Sample DDRs, for $y_R / y_I = 0.1$, and $n = 0.5, 0.8, 1.3$ (shown as open circles in panel B).

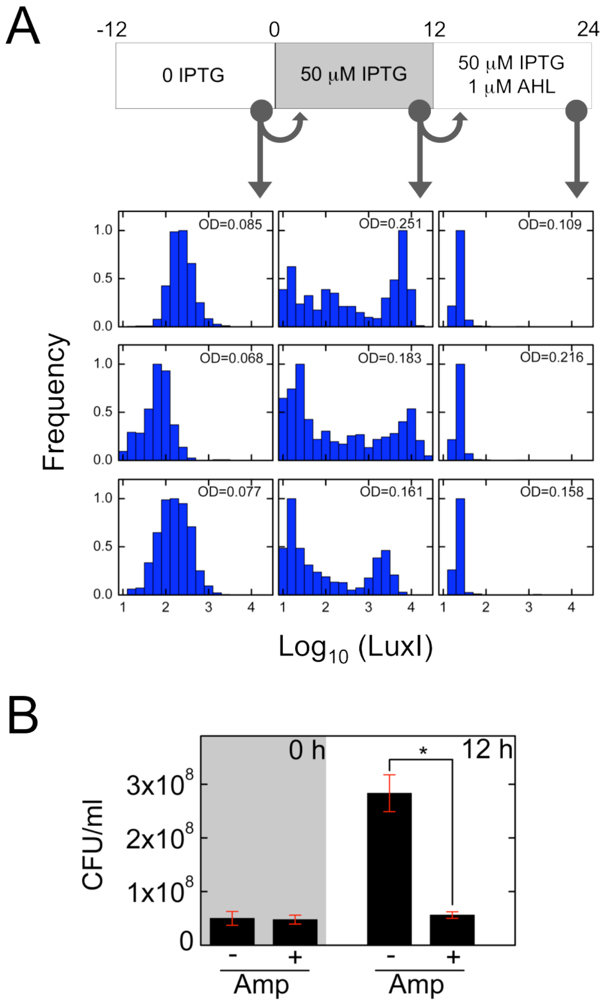

Figure S8: Fluorescence loss measurements. (A) We sampled Aut-IFB cells from various timepoints of the density-dependent protocol (see Materials and Methods: Autonomous loop density-dependent measurements). Cells were extracted for imaging, then re-diluted, just prior to the 0 h, 12 h, and 24 h timepoints; the OD_{600} values indicated correspond to pre-dilution densities. Three replicates of the same experiment are shown. Maximal LuxI::CFP fluorescence values increase throughout the first 12 h growth phase; however, a sub-population of cells show loss of fluorescence. Addition of AHL and subsequent growth to the 24 h timepoint does not lead to fluorescence recovery, indicating that the loss is irreversible. (B) Our constructs are carried on an ampicillin-resistant plasmid backbone. We measured the number of colony-forming units (CFUs) per ml of sample from the 0 h and 12 h extracts, in the presence and absence of ampicillin; errorbars represent standard error of the mean over triplicates. At 0 h all cells are ampicillin resistant (no significant difference between the two counts, $p = 0.89$), while at 12 h the fraction of resistant cells has fallen to less than a fifth ($p = 0.003$), suggesting plasmid loss is responsible for loss of fluorescence.
